# Enhancement of NETosis by ACE2-cross-reactive anti-SARS-CoV-2 RBD antibodies in patients with COVID-19

**DOI:** 10.1186/s12929-024-01026-5

**Published:** 2024-04-18

**Authors:** Kun-Han Hsieh, Chiao-Hsuan Chao, Yi-Ling Cheng, Yen-Chung Lai, Yung-Chun Chuang, Jen-Ren Wang, Sui-Yuan Chang, Yuan-Pin Hung, Yi-Ming Arthur Chen, Wei-Lun Liu, Woei-Jer Chuang, Trai-Ming Yeh

**Affiliations:** 1 Department of Medical Laboratory Science and Biotechnology, College of Medicine, National Cheng Kung University, Tainan, Taiwan; 2 Department of Medical Laboratory and Regenerative Medicine, MacKay Medical College, New Taipei, Taiwan; 3 Department of Immunology and Microbiology, The Scripps Research Institute, La Jolla, CA 92037 USA; 4 Division of Infectious Diseases, Department of Medicine, University of California, San Diego, La Jolla, CA 92037 USA; 5 Leadgene Biomedical, Inc, Tainan, Taiwan; 6 Department of Biomedical Sciences, Chung Shan Medical University, Taichung, Taiwan; 7 Diseases and Vaccinology, National Institute of Infectious National Health Research Institutes, Tainan, Taiwan; 8 Center of Infectious Disease and Signaling Research, National Cheng Kung University, Tainan, Taiwan; 9 Department of Clinical Laboratory Sciences and Medical Biotechnology, College of Medicine, National Taiwan University, Taipei, Taiwan; 10 Department of Laboratory Medicine, National Taiwan University Hospital, Taipei, Taiwan; 11 Department of Internal Medicine, Tainan Hospital, Ministry of Health and Welfare, Tainan, Taiwan; 12 Department of Internal Medicine, National Cheng Kung University, Medical College and Hospital, Tainan, Taiwan; 13 Laboratory of Important Infectious Diseases and Cancer, Department of Medicine, School of Medicine, Fu Jen Catholic University, New Taipei City, 242 Taiwan; 14 School of Medicine, Fu Jen Catholic University, New Taipei City, 242 Taiwan; 15 Diseases and Vaccinology, National Institute of Infectious National Health Research Institutes, Miaoli County, 350 Taiwan; 16 Department of Critical Care Medicine, Fu Jen Catholic University Hospital, Fu Jen Catholic University, New Taipei City, 243 Taiwan; 17 Data Science Center, College of Medicine, Fu Jen Catholic University, New Taipei City, 242 Taiwan; 18 Department of Biochemistry and Molecular Biology, College of Medicine, National Cheng Kung University, Tainan, Taiwan

**Keywords:** COVID-19, Anti-ACE2 autoantibody, NETosis, Cross-reactivity, Thrombosis

## Abstract

**Background:**

High levels of neutrophil extracellular trap (NET) formation or NETosis and autoantibodies are related to poor prognosis and disease severity of COVID-19 patients. Human angiotensin-converting enzyme 2 (ACE2) cross-reactive anti-severe acute respiratory syndrome coronavirus 2 spike protein receptor-binding domain (SARS-CoV-2 RBD) antibodies (CR Abs) have been reported as one of the sources of anti-ACE2 autoantibodies. However, the pathological implications of CR Abs in NET formation remain unknown.

**Methods:**

In this study, we first assessed the presence of CR Abs in the sera of COVID-19 patients with different severity by serological analysis. Sera and purified IgG from CR Abs positive COVID-19 patients as well as a mouse monoclonal Ab (mAb 127) that can recognize both ACE2 and the RBD were tested for their influence on NETosis and the possible mechanisms involved were studied.

**Results:**

An association between CR Abs levels and the severity of COVID-19 in 120 patients was found. The CR Abs-positive sera and IgG from severe COVID-19 patients and mAb 127 significantly activated human leukocytes and triggered NETosis, in the presence of RBD. This NETosis, triggered by the coexistence of CR Abs and RBD, activated thrombus-related cells but was abolished when the interaction between CR Abs and ACE2 or Fc receptors was disrupted. We also revealed that CR Abs-induced NETosis was suppressed in the presence of recombinant ACE2 or the Src family kinase inhibitor, dasatinib. Furthermore, we found that COVID-19 vaccination not only reduced COVID-19 severity but also prevented the production of CR Abs after SARS-CoV-2 infection.

**Conclusions:**

Our findings provide possible pathogenic effects of CR Abs in exacerbating COVID-19 by enhancing NETosis, highlighting ACE2 and dasatinib as potential treatments, and supporting the benefit of vaccination in reducing disease severity and CR Abs production in COVID-19 patients.

**Supplementary Information:**

The online version contains supplementary material available at 10.1186/s12929-024-01026-5.

## Background

The coronavirus disease 2019 (COVID-19) pandemic, which is caused by severe acute respiratory syndrome coronavirus 2 (SARS-CoV-2) infection, has led to considerable morbidity and mortality worldwide; it has caused a staggering number of deaths, reaching seven million deaths to date [1].

The receptor-binding domain (RBD) of the S1 subunit of the SARS-CoV-2 spike protein plays a key role in binding to the angiotensin-converting enzyme 2 (ACE2) receptor [2]. To control the COVID-19 pandemic, vaccines that target the spike protein have been developed and deployed to induce the production of neutralizing antibodies and prevent infection and the spread of disease [3]. Even though infection still occurs in some individuals after vaccination, the risk of severe illness and death is reduced [4].

Most COVID-19 patients present mild clinical symptoms, such as fever, chills, and typical respiratory compromise [5]. However, in severe cases, a systemic hyperimmune response occurs, which can lead to cytokine storms, thrombus deposition, and vascular dysfunction that leads to acute respiratory failure, organ dysfunction and death [6]. Among the factors that cause the dysregulation of immune responses in COVID-19, neutrophil activation, especially the release of neutrophil extracellular traps (NETs), has attracted substantial attention [7, 8]. NETs, which contain DNA molecules and granule-derived enzymes such as myeloperoxidase (MPO), are released from neutrophils through a process called NETosis. Activation of protein arginine deiminase 4 (PAD-4) plays an essential role in NET formation by mediating histone hypercitrullination and chromatin decondensation and facilitating the expulsion of DNA from neutrophils [9, 10]. Typically, NETs are part of the innate immune response; they can capture and kill extracellular pathogens to prevent their spread during infection, and they can be cleared by DNase enzymes. However, abundant NETs have been observed in lesions of lung biopsies from patients with severe COVID-19, and high levels of free NETs have been observed in the sera of hospitalized patients [7, 11, 12]. In addition, excess NET formation is related to poor prognosis and COVID-19 severity. Therefore, NETs are considered key factors that cause immunothrombosis in COVID-19 [13, 14]. To better address this problem, there is an urgent need to understand the mechanisms of NET formation in COVID-19.

Another hallmark of hyperimmune responses in severe COVID-19 patients, is the production of autoantibodies against various self-antigens [15, 16]. Among the series of autoantibodies, autoantibodies against the SARS-CoV-2 receptor ACE2 have been observed after SARS-CoV-2 infection [17]. In addition, the serum levels of anti-ACE2 autoantibodies are associated with COVID-19 disease severity [18, 19]. Interestingly, we previously showed that anti-RBD antibodies that can cross-react with ACE2 (CR Abs) are one of the sources of anti-ACE2 autoantibodies in COVID-19 [20]. In addition, an ACE2-cross-reactive anti-RBD monoclonal antibody (mAb 127) has been isolated from RBD-immunized mice [20]. Nevertheless, the pathological functions of CR Abs remain unclear.

In this study, we demonstrated a significant increase in the levels of both anti-ACE2 and CR Abs in the sera of patients with severe COVID-19. We used mAb 127 to elucidate the relationship between CR Abs and hyperinflammation in COVID-19 by demonstrating that mAb 127 induced NETosis by binding to both ACE2 and the Fc receptor of neutrophils. Importantly, we used both sera containing CR Abs and purified CR IgG from patients to confirm that CR Abs enhanced NETosis. Furthermore, blocking the binding of CR Abs by incubation with recombinant human ACE2 or inhibiting NETosis with the Src inhibitor dasatinib effectively prevented NET formation and subsequent formation of microthrombi in vitro. Finally, we observed the prevention of the production of CR Abs in SARS-CoV-2 infection by vaccination, providing a possible explanation for vaccination in reducing the fatality of COVID-19.

## Materials and methods

### Cohort information

The definition of clinical spectrum of SARS-CoV-2 infection followed the COVID-19 treatment guidelines of National Institutes of Health. The mild patients with COVID-19 were defined as those individuals who had any of the various signs and symptoms of COVID-19 (e.g., fever, cough, sore throat, malaise, headache, muscle pain, nausea, vomiting, diarrhea, loss of taste and smell) but did not have shortness of breath, dyspnea, or abnormal chest imaging. The moderate/severe patients with COVID-19 were defined as those individuals who experienced a fever (≧38℃) or respiratory symptoms and subsequently developed pneumonia requiring oxygen therapy or other complications within 14 days (inclusive), leading to hospitalization (including emergency room admission). The vaccinated COVID-19 patients received their vaccinations before being infected with SARS-CoV-2.

### Recombinant protein, monoclonal antibody and F(ab')_2_ fragment generation

RBD recombinant protein (YP_009724390) was expressed by S2 cells and its purification was performed as previously described [21]. The ACE2-cross-reactive anti-RBD (mAb 127) and the anti-RBD mAb (LGSV201), which does not bind to ACE2, were generated from RBD-immunized mice as previously described (Fig. S1) [20]. Recombinant human ACE2 (rACE2) was purchased from Genetex (Irvine, CA). ZMAB-mouse IgG2b (cmIgG) was used as an isotype-matched antibody control (AB Biosciences, Concord, MA). The cmIgG and mAb 127 F(ab')_2_ fragments were prepared using a Pierce™ F(ab’)_2_ Preparation Kit (Thermo Fisher Scientific, Waltham, MA).

### Indirect ELISAs

Diluted sera (1:100 for anti-nucleocapsid or anti-RBD antibodies, 1:50 for anti-ACE2 autoantibodies) or diluted monoclonal antibodies were added to the indicated protein-coated plate (2 µg/mL). The binding antibodies were detected by either goat anti-human IgG-horseradish peroxidase (HRP)-conjugated antibodies (Thermo Fisher Scientific) or goat anti-mouse IgG HRP antibodies (Leadgene Biomedical, Taiwan). For color visualization, the tetramethylbenzidine (TMB) reagent (Clinical Science Products, Mansfield, MA) was added, and the reaction was stopped by 2 N H_2_SO_4_. The absorbance at OD 450 nm was read by a VersaMax microplate reader (Molecular Devices, Sunnyvale, CA).

### Serum preadsorption assay

Diluted sera (1:50 in PBS) were added to bovine serum albumin (BSA) (10 μg/mL, 100 μL)- or RBD (10 μg/mL, 100 μL)-coated wells. After incubation at 37 °C for 1 h, the diluted sera were transferred to wells that were coated with rACE2 (2 μg/mL, 100 μL). Bound anti-ACE2 IgG was detected using HRP-conjugated goat anti-human IgG antibodies (1:4,000) (Thermo Fisher Scientific). Positive anti-ACE2 autoAb is determined by the OD of sera binding to ACE2-coated ELISA plates when exceeding 0.39, as determined by the cutoff value (> 2.1-fold of OD values of anti-ACE2 IgG in unvaccinated healthy donors). Positive CR Ab was determined by the OD of sera binding to ACE2-coated ELISA plates when (OD of BSA preadsorption)-(OD of RBD preadsorption) > 0.1.

### Isolation of human leukocytes, peripheral blood mononuclear cells, neutrophils, and platelets

Whole blood was collected in EDTA vacutainers and centrifuged to obtain buffy coats. The cells were subjected to RBC lysis and washed with PBS to isolate human leukocytes. Peripheral blood mononuclear cells (PBMCs) were isolated from the buffy coats by density gradient centrifugation using Histopaque-1077 (Sigma‒Aldrich, St. Louis, MO). The purity of the PBMCs was confirmed by flow cytometry (CytoFLEX S, Beckman Coulter, Indianapolis, IN) using an anti-human CD14 antibody (sc-1182, Santa Cruz Biotechnology, Santa Cruz, CA). Neutrophils were isolated from the buffy coats by density gradient centrifugation using Polymorphprep™ (ProteoGenix, Schiltigheim, France). The purity and viability of the neutrophils were confirmed by flow cytometry (CytoFLEX S) using an anti-human CD11b antibody (F-2648, Sigma‒Aldrich) and 7-AAD (BD Pharmingen, La Jolla, CA). Platelets were isolated from human whole blood that was collected in ACD vacutainers by centrifugation at 200 × g for 20 min. Platelet-rich plasma was obtained and then further centrifuged at 800 × g with 100 nM prostaglandin E1 for another 20 min. The platelet pellets were suspended in Tyrode’s buffer supplemented with 100 nM PGE1. The purity of the isolated platelets was determined by measuring surface CD61 expression (GTX61848, Genetex) using flow cytometry (CytoFLEX S).

### In vitro human leukocyte stimulation

Human isolated leukocytes, PBMCs and neutrophils (2 × 10^6^ cells/mL) were seeded in 48-well tissue culture plates and treated with the indicated conditions. Notably, to reduce potential interference from various components in the serum, we employed a preadsorption model for stimulation when treating cells with serum samples. Briefly, cells were preabsorbed with diluted sera in the presence or absence of ACE2 at 37 °C for 30 min. Then, the cells were centrifuged at 500 × g for 5 min to remove the supernatant and resuspended in RBD-containing medium. To inhibit mAb 127-induced NETosis, neutrophils were incubated with mAb 127 and RBD and cotreated with inhibitors (dasatinib, SML-2589; compound C, 171260; Ly294002, 19–142; Cl-Amidine, 50652, Sigma‒Aldrich). The levels of tumor necrosis factor-α (TNF-α), interleukin 1β (IL-1β), IL-6, and IL-8) and MPO in the supernatants were quantified by ELISA (R&D Systems Inc., Minneapolis, MN), and the degree of adhesion or NET formation was measured by immunofluorescence staining.

### Neutrophil adhesion assay

The degree of neutrophil adhesion was determined as previously described [22]. After the indicated treatments, nonadherent neutrophils were removed by washing twice with PBS. The adherent cells were fixed with 4% paraformaldehyde and permeabilized with 0.5% Triton X-100 in PBS. The number of adherent cells and the expression of a human granulocyte activation marker (CD66b) were detected by a mouse anti-human CD66b antibody (GTX19779, GeneTex) and Hoechst 33,342 (Invitrogen, Carlsbad, CA) and visualized by fluorescence microscopy (Olympus FluoView FV1000, Japan) and quantified using ImageJ.

### Quantification of NET formation

The degree of NET formation was determined as previously described [23]. Isolated neutrophils under the indicated treatment were spun onto slides by a cytocentrifuge. After fixation and permeabilization, slides were incubated with mouse anti-human CD66b antibody (GTX19779, GeneTex) or rabbit anti-human citrullinated histone antibody (ab5103, Abcam, Cambridge, United Kingdom) and rabbit or mouse anti-human MPO antibody (A1374, ABclonal; GTX75318, GeneTex) and Hoechst 33,342. The degree of NET formation was determined by quantifying the overlap of CD66b or histones and MPO and nuclei using fluorescence microscopy (Olympus FluoView FV1000) and ImageJ.

### NET purification and treatment with NET-containing supernatants

Isolated human neutrophils (5 × 10^6^ cells/mL) were treated with 500 nM phorbol 12-myristate 13-acetate (PMA) for 4 h, and the NETs were purified as previously described [24]. Purified NETs were used at a concentration of 1 μg/mL as a positive control treatment. For the supernatant treatment, isolated human PBMCs, human umbilical vein endothelial cells (HUVECs) or isolated human platelets were incubated with the indicated conditioned medium and RPMI medium at a ratio of 1:2. PBMCs and HUVECs were stimulated for 24 h, while platelets were stimulated for 15 min.

### Endothelial cell permeability assay

HUVECs were grown in Transwell plates (0.4 μm; Corning B.V. Life Sciences, the Netherlands) with 10% fetal bovine serum (FBS)-containing EGM-2 medium until a monolayer was formed. The upper chambers were reconstituted with the indicated conditioned media and RPMI medium at a ratio of 1:2 or with EGM-2 medium alone. After 24 h of incubation, the media in the upper chambers were replaced with serum-free media containing streptavidin-HRP (1:100, R&D Systems). After 15 min, medium from the lower chamber was collected, and HRP activity was measured by adding TMB reagent (Clinical Science Products). Color development was detected by a VersaMax microplate reader (Molecular Devices) at 450 nm.

### Flow cytometry analysis of platelet activation

Platelet activation was determined by measuring P-selectin (CD62P) surface expression. After the indicated treatment, isolated human platelets (1 × 10^7^ cells/100 μl) were incubated with FITC-conjugated anti-human CD62P (BD Bioscience, San Diego, CA) or FITC-conjugated isotype-matched antibodies (BD Bioscience) and analyzed using flow cytometry (CytoFLEX S).

### HUVEC, neutrophil and platelet coculture model

In a 24-well plate, confluent HUVECs on coverslips were incubated with a total of 5 × 10^5^ neutrophils and 1 × 10^7^ platelets, followed by treatment with the recombinant RBD protein in the presence or absence of the indicated antibodies (mAbs 127, LGSV201, or isotype cmIgG) or dasatinib for 3 h. The cells were washed twice with PBS/2% FBS, fixed with 4% paraformaldehyde, and permeabilized with 0.1% Triton X-100. After blocking, the cells were incubated with a rabbit anti-human CD61 monoclonal antibody (GTX61848, Genetex) and a mouse anti-human CD66b monoclonal antibody (GTX19779, Genetex) and then incubated with fluorescently labeled antibodies against rabbit or mouse IgG (A-11017, A-11072, Invitrogen) and Hoechst 33342 for 1 h and visualized by fluorescence microscopy (Olympus FluoView FV1000, Japan).

### Purification and preadsorption of immunoglobulin G (IgG) from COVID-19 patient serum

Human total IgG in the collected sera from COVID-19 patients or healthy donors (100 µl) was purified using Pierce Protein G Plus Agarose (Thermo Fisher Scientific). Elution was performed with 0.1 M glycine–HCl (pH 2.7) and immediately neutralized with neutralizing buffer (1 M Tris–HCl, pH 9.0). Purified human IgG underwent an exchange with PBS through Amicon Ultra filters with a 30 kDa molecular weight cutoff (Sigma‒Aldrich). To confirm that the concentration of purified total human IgG retained the characteristic features of CR Abs for neutrophil treatment, a range of 50–200 µg/mL of purified human IgG from different individuals was tested using both indirect ELISA and BSA- or RBD-preadsorption ELISA. For IgG treatment from COVID-19 patients, human isolated neutrophils underwent preadsorption with 100 µg/mL purified human IgG in the presence or absence of ACE2 at 37 °C for 30 min. Subsequently, the cells were centrifuged at 500 × g for 5 min to eliminate the supernatant and cultured in 10 µg/mL RBD-containing medium at 37 °C for 24 h. For inhibitor treatment, neutrophils were incubated with 10 µg/mL RBD and cotreated with 50 nM dasatinib at 37 °C for 24 h. The levels of IL-8 and MPO in the supernatants were quantified by ELISA (R&D Systems Inc.), and the degree of adhesion or NET formation was measured by immunofluorescence staining.

### Statistical analysis

All statistical analyses were conducted using either an unpaired or paired Student’s t test for comparisons between two independent groups. One-way ANOVA or two-way ANOVA and post hoc Tukey tests were used for multigroup comparisons. Prism software (GraphPad Software Inc., CA) was utilized for statistical analysis. The data are presented as the means ± standard deviations (S.Ds.) from at least three independent experiments. *P < 0.05, **P < 0.01, ***P < 0.001, ****P < 0.0001 and ns indicates no significance based on 95% two-tailed confidence intervals.

## Results

### Increased serum levels of ACE2-cross-reactive anti-RBD antibodies in moderate/severe COVID-19 patients’ sera may induce stronger leukocyte activation in vitro

**Fig. 1:**
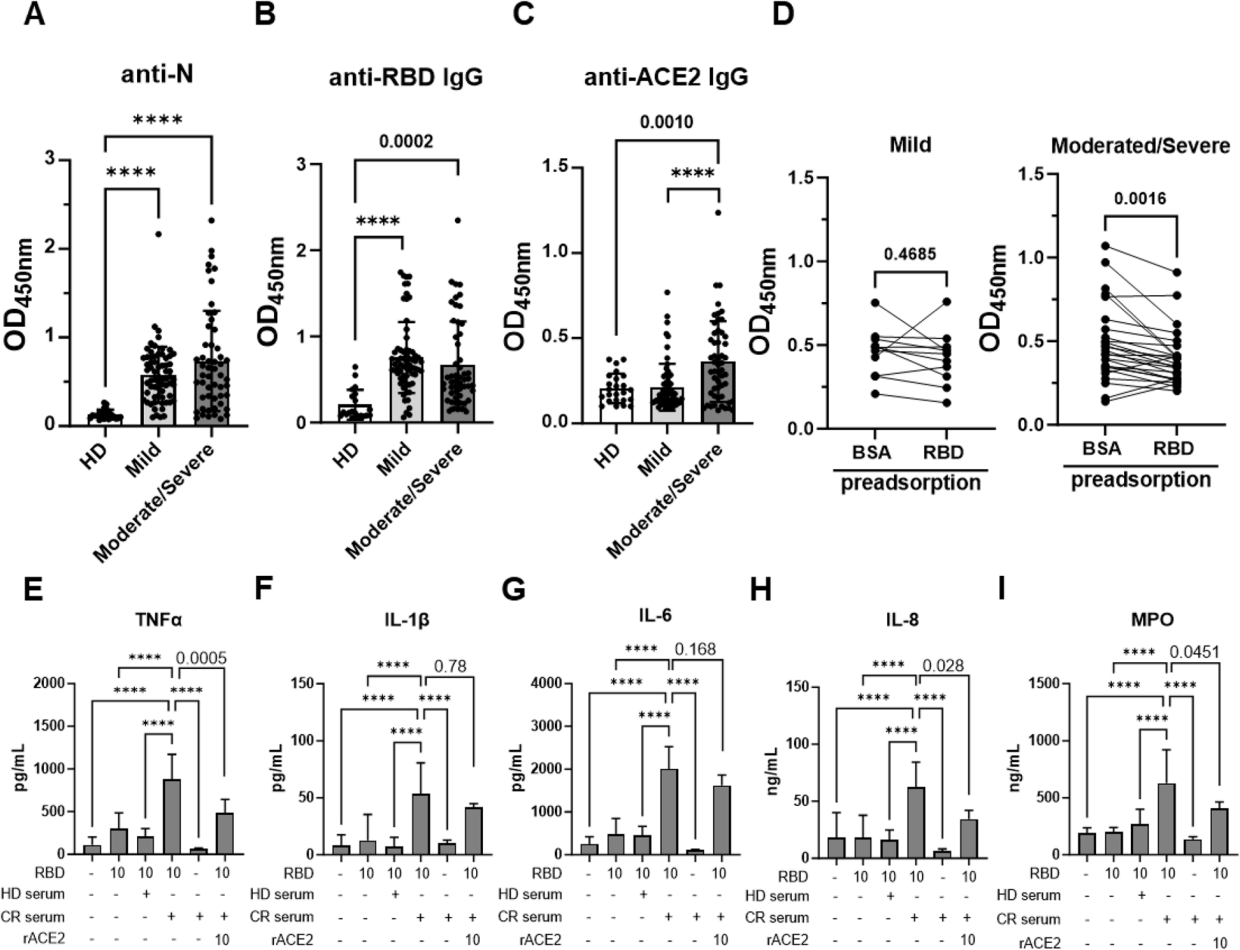
Significant increase of CR Abs IgG in moderate/severe COVID-19 patients’ sera may contribute to cytokine secretion and NET formation in human leukocytes in the presence of RBD. IgG against nucleocapsid, N, RBD and ACE2 proteins in sera from COVID-19 patients and healthy donors (HD) were measured by ELISA. Comparison of (**A**) the levels of IgG against N, (**B**) RBD, and (**C**) ACE2 in mild vs. moderate/severe COVID-19 patients. (**D**) Anti-ACE2 IgG levels in sera of mild vs. moderate/severe COVID-19 patients after BSA or RBD preadsorption. For the leukocyte activation test, isolated human leukocytes were preincubated with 1:100 diluted HD serum or sera from CR Ab-positive COVID-19 patients (CR serum) as indicated in the presence or absence of additional rACE2 (10 μg/mL) for 30 min. Afterward, unbound antibodies were removed by centrifugation, and the cells were treated with the RBD protein (10 μg/mL) for 24 h. The supernatants were collected to measure the (**E**) TNF-α, (**F**) IL-1β, (**G**) IL-6, (**H**) IL-8, and (**I**) MPO levels using ELISA kits. The averages of triplicate cultures ± SD are shown. Multiple comparison of antibody response and leukocyte activation tests (*n* = 3–5) was conducted by one-way ANOVA and Tukey’s post hoc test. The comparison between BSA and RBD preadsorption was analyzed by paired Student’s t-test; *P* values were displayed, and for values less than 0.0001, they were represented as ****

To understand whether ACE2-cross-reactive anti-RBD antibodies, referred to as CR Abs, are associated with the progression of COVID-19, we stratified the patients based on disease severity for serological analysis (Supplementary Table 1). First, we confirmed that the levels of anti-N IgG were significantly higher in the COVID-19 patient group than in the healthy donor group, indicating a natural infection (Fig. 1A). In comparison of healthy donors, the levels of anti-RBD IgG in both the mild and moderate/severe COVID-19 patient groups were obviously elevated (Fig. 1B). However, only a significant increase in the anti-ACE2 IgG levels in the moderate/severe patient group was observed (Fig. 1C). To further confirm whether these anti-ACE2 IgG antibodies encompassed CR Abs, sera from COVID-19 patients with OD values of anti-ACE2 IgG exceeding 0.39, as determined by the cutoff value (> 2.1 fold of OD values of anti-ACE2 IgG in unvaccinated individuals), were selected for BSA or RBD preadsorption ELISAs. We discovered notably increased CR Abs in the moderate/severe patient group but not the mild patient group (Fig. 1D), suggesting a potential pathogenic role of CR Abs in the severity of COVID-19.

To explore the contribution of CR Abs to hyperinflammation in COVID-19, we applied CR Ab-containing sera from COVID-19 patients with severe disease to isolated leukocytes, the primary cells expressing ACE2 in circulating blood (Fig. S2). To minimize potential interference from various serum components, isolated human leukocytes or neutrophils were preincubated with diluted serum for 30 min. Then, unbound substances were removed by washing before adding RBD recombinant protein, purified from insect cells (S2) expression system, for a 24 h incubation. Notably, RBD alone did not trigger significant cytokine secretion nor NET formation, confirming the absence of lipopolysaccharide contamination in the recombinant protein (Fig. 1E-I and Fig. S3, Fig. S4). Compared to healthy controls, preincubation of CR Ab-containing sera at a 1:100 dilution resulted in significant increases in NET formation and TNF-α, IL-1β, IL-6, IL-8 and MPO secretion by isolated human leukocytes after 24 h of RBD treatment (Fig. 1E-I and Fig. S3). NET formation and the secretion of TNF-α, IL-8 and MPO, except for IL-1β and IL-6, which were induced by preincubation with CR Ab-containing sera, were significantly blocked when human leukocytes were preincubated with ACE2. Furthermore, these responses were observed only in the presence of RBD, as CR Ab-containing sera alone did not exert these effects (Fig. 1E-I). Consistently, CR Abs from RBD-immunized mice (mAb 127), but not isotype control mouse IgG, cmIgG, with the RBD significantly increased TNF-α, IL-1β, IL-6 and IL-8 secretion from isolated human leukocytes (Fig. S4A-S4D). Altogether, our results support that CR Abs are positively correlated with the deterioration of COVID-19, and interacting with RBD and ACE2 on the cell surface is needed for CR Abs to trigger the inflammatory response and NETosis of leukocytes.

### ACE2-cross-reactive anti-RBD antibodies induce neutrophil activation and NETosis in the presence of RBD

**Fig. 2:**
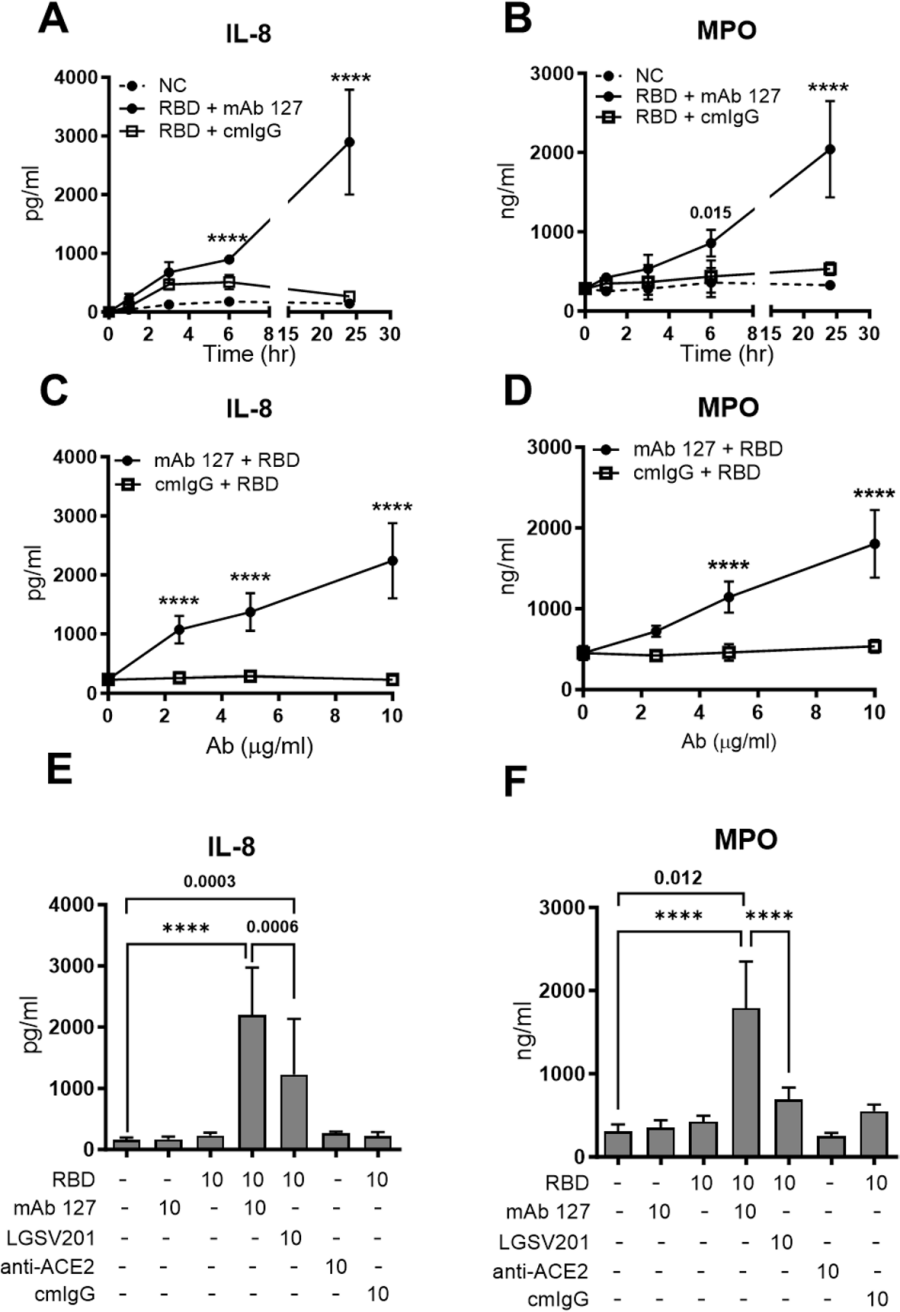
CR Abs induce human neutrophils to secrete IL-8 and MPO in the presence of RBD. (**A**, **B**) Isolated human neutrophils (2 × 10^6^ cells/mL, 0.3 mL/test) were treated with the recombinant RBD protein in the presence or absence of the indicated antibodies (mAb 127 or cmIgG) for different time points (1, 3, 6, and 24 h). (**C**, **D**) Neutrophils were cotreated with 10 μg/mL RBD recombinant protein and mAb 127 or cmIgG at different concentrations (2.5, 5, or 10 μg/mL). (**E**, **F**) Neutrophils were stimulated with the recombinant RBD protein in the presence or absence of the indicated antibodies (mAbs 127, LGSV201, cmIgG, or anti-ACE2) for 24 h. The supernatants from these experiments were collected, and IL-8 and MPO levels were quantified using ELISA kits. The averages of triplicate cultures ± SD are shown. Statistical significance was calculated using one-way ANOVA and Tukey’s post hoc test; *P* values were displayed, and for values less than 0.0001, they were represented as ****

We isolated human neutrophils and peripheral blood mononuclear cells (PBMCs) from human blood to further clarify which leukocyte cells play a major role in the CR Abs-induced inflammatory response. Western blotting analysis revealed that neutrophils were the primary cells among leukocytes that expressed ACE2 (Fig. S5). Consistently, in the presence of the RBD, mAb 127 (but not cmIgG) significantly induced the secretion of IL-8 and MPO from neutrophils in a time-dependent (1, 3, 6, or 24 h) and antibody dose-dependent (2.5, 5, or 10 μg/mL) manner (Fig. 2A-2D). However, there was no detectable release of TNF-α, IL-1β and IL-6 when neutrophils were stimulated with mAb 127 or LGSV201 (mAb that can only recognize RBD, used as an RBD-IgG immunocomplex control, Fig. S1) in the presence of RBD (Fig. S6A-S6C). On the other hand, TNF-α and IL-6 secretion was induced when PBMCs were treated with mAb 127 or LGSV201 in the presence of the RBD (Fig. S6D and S6E). Since PBMCs lack ACE2 expression, these results suggest that binding to Fc receptor on PBMCs by RBD-IgG immunocomplex can induce TNF-α and IL-6 secretion. Similarly, both mAb 127 and LGSV201 significantly induced IL-8 and MPO secretion from neutrophils compared to the negative control (Fig. 2E and F). However, mAb 127 induced significantly higher degrees of IL-8 and MPO secretion from neutrophils than LGSV201. On the other hand, like cmIgG, polyclonal anti-ACE2 antibodies that could bind to ACE2 on the cell membrane could not induce neutrophils to secrete IL-8 and MPO (Fig. 2E and F).

**Fig. 3:**
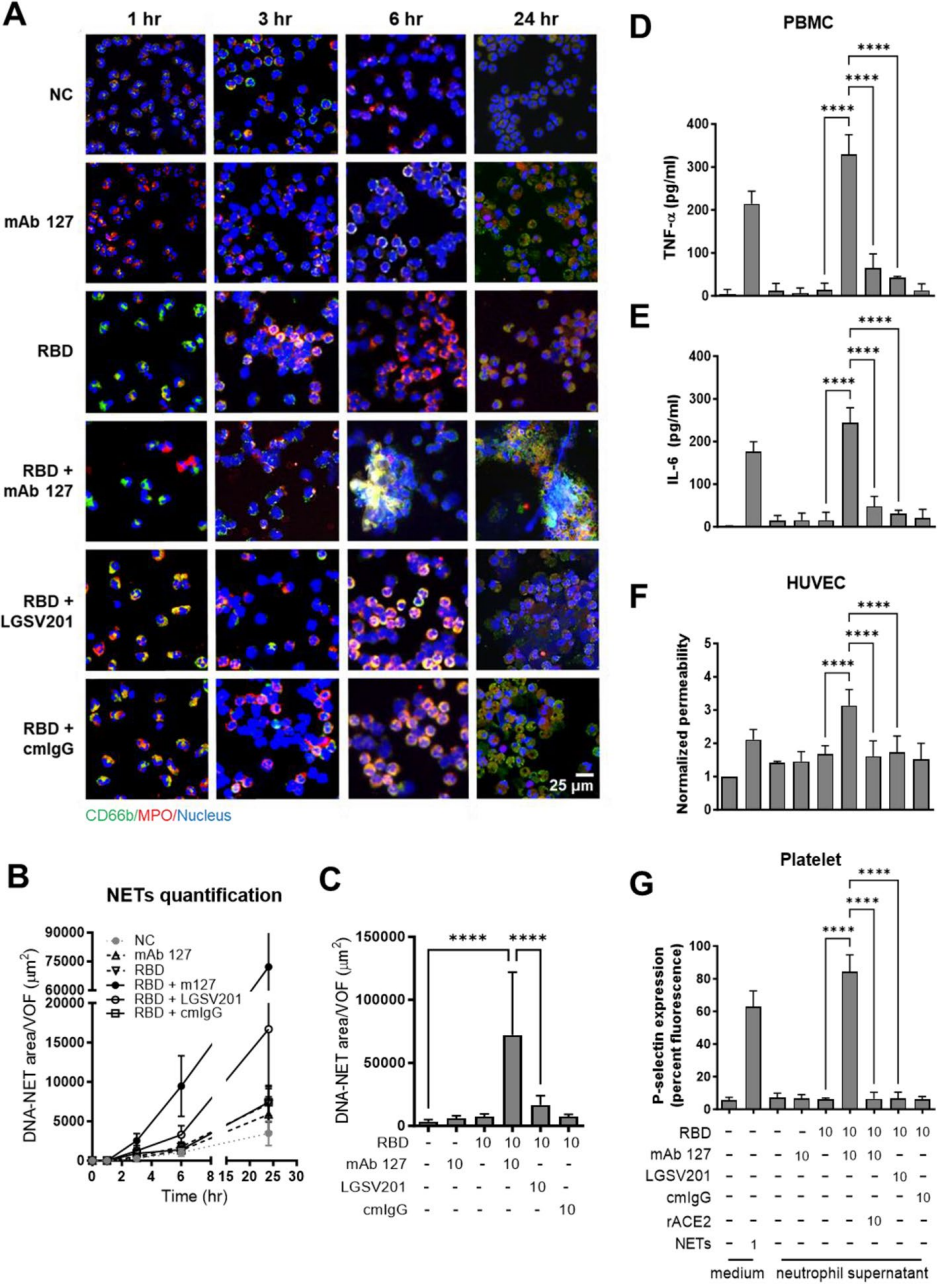
NETosis triggered by CR Abs in the presence of RBD drives thrombosis-associated cell activation. (**A**) Isolated human neutrophils were treated with 10 μg/mL recombinant RBD protein and the indicated antibodies (mAbs 127, LGSV201, or cmIgG, 10 μg/mL) or not. After the indicated time points, the cell suspensions were spun onto a microscope slide by using a cytocentrifuge, fixed and stained with anti-CD66b antibody (green), anti-MPO antibody (red) and DAPI (blue) nuclear stain and then visualized using immunofluorescence staining. The area of NET formation at (**B**) different time points or (**C**) 24 h after stimulation was quantified by ImageJ. Views for NET quantification were randomly selected with 9 pictures from each experiment. The supernatants of neutrophils were harvested after different treatments and further administered to isolated human PBMCs, HUVECs, or platelets. Purified PMA-induced NETs (1 μg/mL) were used as the positive control, and in some experiments, rACE2 was added. Following 24 h of stimulation with neutrophil-conditioned media, PBMC supernatants were collected to measure the (**D**) TNF-α and (**E**) IL-6 levels using ELISA kits. (**F**) Endothelial barrier integrity was determined by a Transwell permeability assay, as described in the Materials and Methods. (**G**) After 15 min of stimulation, the percent fluorescence of P-selectin surface expression on platelets was measured by anti-CD62p FITC-conjugated antibodies and further analyzed by cytoFLEX. The averages of triplicate cultures ± SD are shown. Statistical significance was calculated using one-way ANOVA and Tukey’s post hoc test, ****p < 0.0001. Bar: 25 μm

In addition to secreting IL-8 and MPO, activated neutrophils undergo morphological changes. When stimulated with the RBD alone, neutrophils adhered to the culture plate and exhibited lamellipodium-like structures with elevated CD66b expression after 1 h of stimulation, as shown in Fig. S7A. However, these morphological changes in activated neutrophils were more dramatic in the mAb 127 cotreated group than in the LGSV201 and cmIgG cotreated groups. Indeed, mAb 127 treatment in the presence of RBD significantly increased the number of adherent neutrophils and the expression of CD66b compared to the other groups (Fig. S7B and S7C). Nevertheless, the levels of adhesion and CD66b expression of activated neutrophils that were treated with RBD in the presence or absence of different mAbs decreased over time after the peak at 1 h (Fig. S7A). Moreover, NET formation was observed only in the groups that were cotreated with RBD and mAb 127 after 6 h of stimulation (Fig. 3A). As shown in Fig. 3B and C, NET production was significantly higher in the group that was cotreated with RBD and mAb 127 at 24 h than in the other groups. Collectively, these results imply that mAb 127 mainly augmented the immune response by utilizing cross-reactivity with ACE2 to target neutrophil activation and promoted NETosis over a long period.

### NETs from activated neutrophils drive thrombosis-associated cell activation in vitro

Since NETs are regarded as a trigger in the development of complications in COVID-19, we tested whether mAb 127-induced NETosis gives rise to thrombosis by collecting conditioned media from neutrophils after 24 h of exposure to different treatments, as described above, and administering these media to thrombosis-associated cells, including PBMCs, human umbilical vein endothelial cells (HUVECs) and platelets. Purified NETs from neutrophils that were treated with 1 μg/mL PMA were used as the positive control. Cytokine level quantification by ELISA, endothelial cell permeability assays, and platelet adhesion marker analysis by flow cytometry (CytoFLEX S) were performed to measure PBMC, HUVEC and platelet activation, respectively. The results showed that only the supernatants of neutrophils that were stimulated with mAb 127 and the RBD significantly induced the activation of these three types of cells compared to the other groups (Fig. 3D-G). Furthermore, when neutrophils were stimulated with mAb 127 and the RBD in the presence of rACE2, the effects of the neutrophil supernatants on stimulating these cells were all abolished (Fig. 3D-G), suggesting that ACE2 participated in CR Ab-induced NETs.

### Both ACE2 and the Fc receptor are needed for ACE2-cross-reactive anti-RBD antibodies to trigger NETosis

**Fig. 4:**
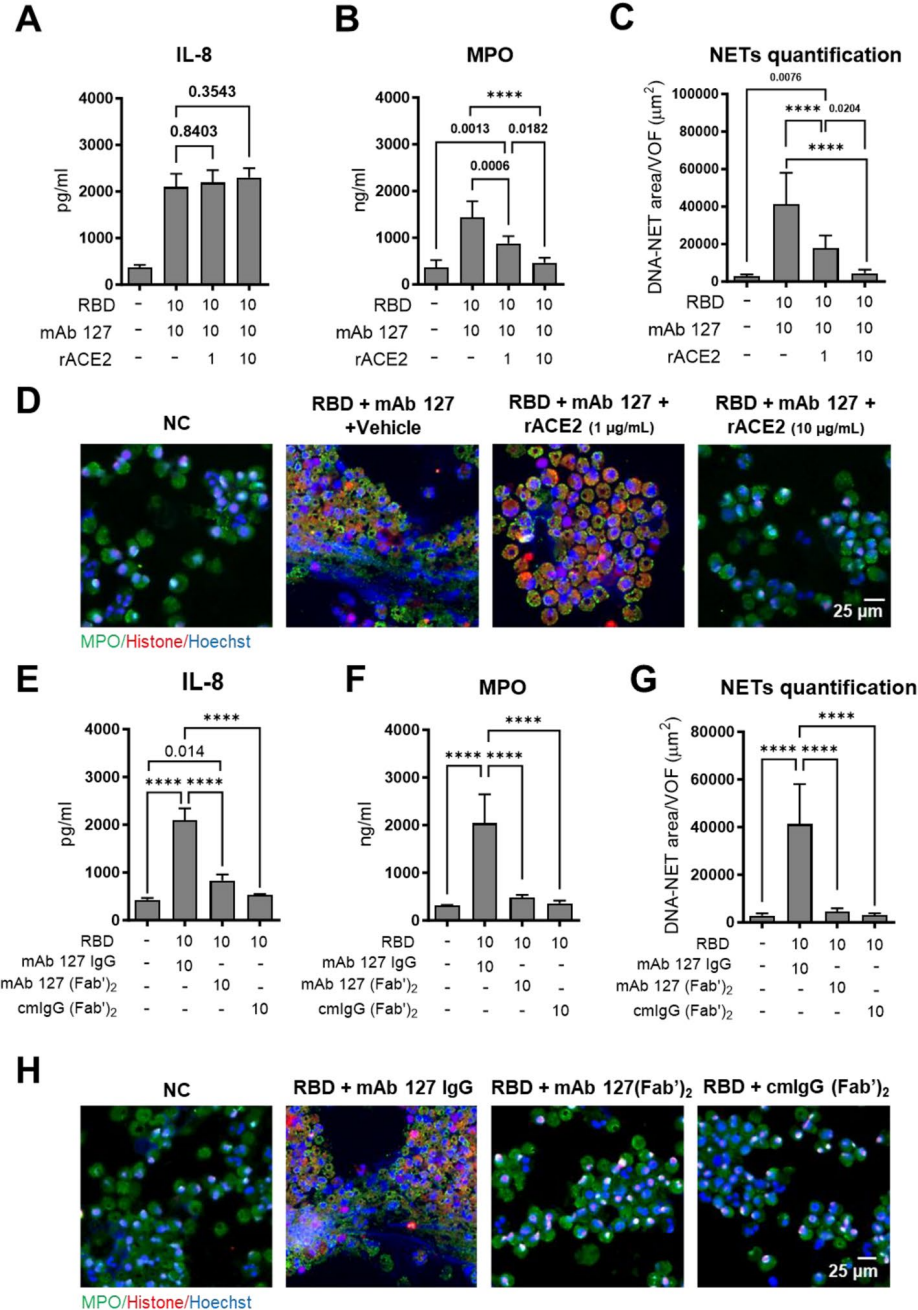
CR Abs-triggered NETosis is dependent on ACE2 and the Fc receptor. Isolated human neutrophils were treated with recombinant RBD protein (10 μg/mL) and the indicated antibodies (mAb 127, mAb 127-F(ab’)_2_, or cmIgG-F(ab’)_2_, 10 μg/mL) in the presence or absence of additional rACE2 (1 or 10 μg/mL) for 24 h. The supernatants were collected to measure the levels of (**A**, **E**) IL-8 and (**B**, **F**) MPO using ELISA kits. (**C**, **D**, **G**, **H**) The cell suspensions were spun onto a microscope slide by using a cytocentrifuge and were fixed and subjected to immunofluorescence staining with an anti-MPO antibody (green), anti-histone antibody (red) and Hoechst (blue) nuclear stain, and the area of NET formation was quantified by ImageJ. The averages of triplicate cultures ± SD are shown. The views for NET quantification were randomly selected with 9 pictures from each experiment. Statistical significance was calculated using one-way ANOVA and Tukey’s post hoc test; *P* values were displayed, and for values less than 0.0001, they were represented as ****. Bar: 25 μm

To further investigate whether ACE2 plays a crucial role in mAb 127-induced pathological effects, we preincubated mAb 127 with rACE2 at concentrations of 1 or 10 μg/mL for 1 h and then treated neutrophils with the RBD. Interestingly, while rACE2 could not suppress mAb 127-induced IL-8 secretion by activated neutrophils (Fig. 4A), it effectively inhibited the MPO secretion and NET formation induced by mAb 127 and the RBD (Fig. 4B, C). In addition, immunofluorescence staining confirmed that neutrophil NETosis induced by mAb 127 and the RBD was inhibited in the presence of rACE2 in a dose-dependent manner (Fig. 4D). To further investigate the contribution of Fc receptors to mAb 127-induced NETosis, the cells were costimulated with mAb 127-F(ab')_2_ or cmIgG-F(ab')_2_ and RBD at 10 μg/mL. Compared to the mAb 127-treated group, all three markers we measured (IL-8, MPO and NETs) were significantly decreased in the mAb 127-F(ab')_2_-treated group (Fig. 4E-G). Immunofluorescence microscopy images also confirmed a reduction in the NET area in the mAb 127-F(ab')_2_-treated group (Fig. 4H). Altogether, these results suggested that both ACE2 and Fc receptors were needed for mAb 127 to trigger neutrophil activation and NETosis in vitro.

### Src- and PAD-4-dependent pathways are involved in ACE2-cross-reactive anti-RBD antibody-induced NETosis in the presence of RBD

**Fig. 5:**
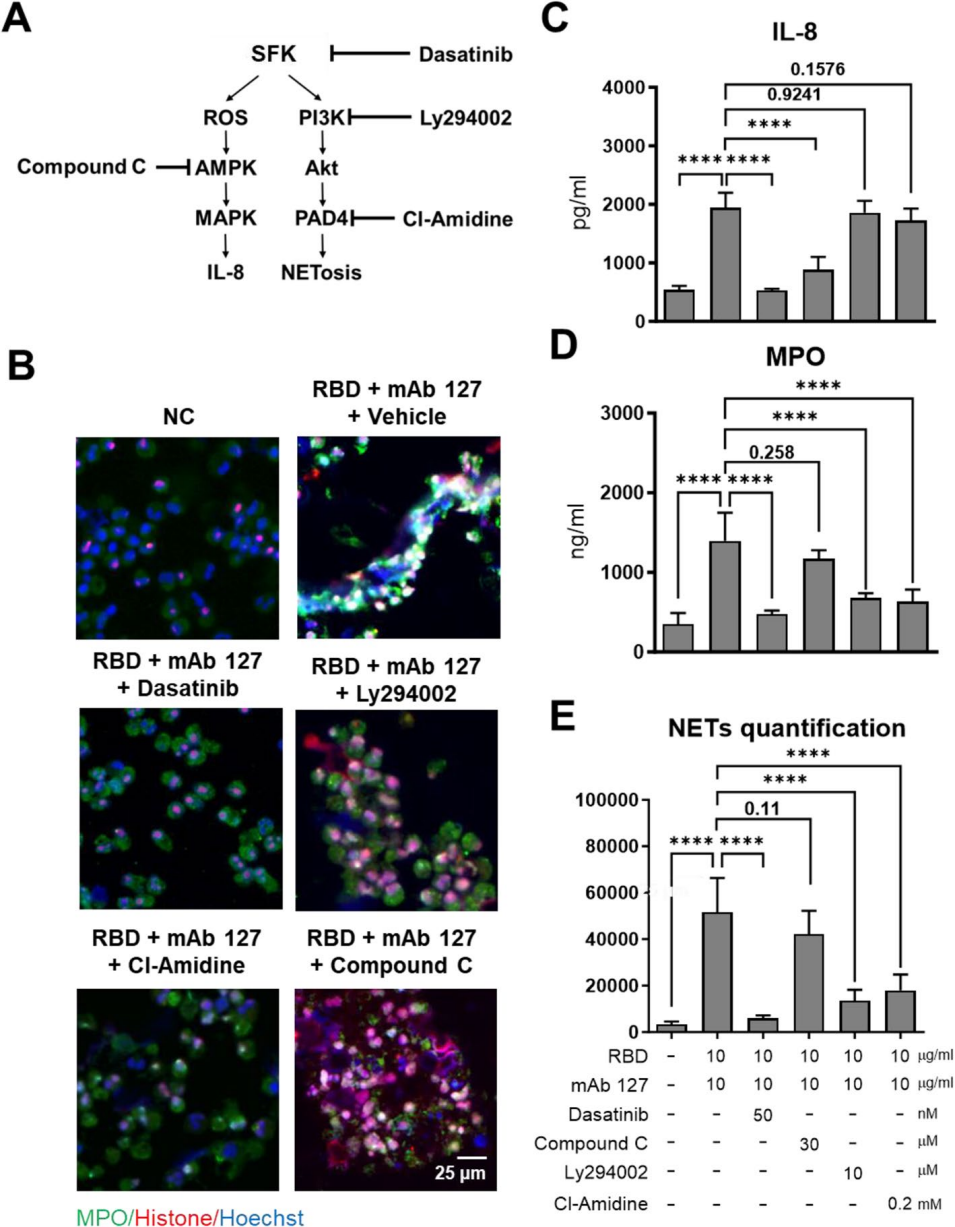
SFK and PAD-4 signaling is involved in CR Abs-triggered NETosis in vitro. (**A**) The picture describes the SFK-induced signaling pathway by which IL-8 is produced/secreted or NETs are formed by neutrophils and the target protein of each inhibitor. Isolated human neutrophils were stimulated with mAb 127 (10 μg/mL) and the RBD protein (10 μg/mL) and cotreated in the presence or absence of the indicated inhibitor at the indicated concentrations for 24 h (vehicle control, DMSO; Compound C, an AMPK inhibitor, 30 μM; dasatinib, a src protein family inhibitor, 50 nM; Ly294002, a PI3K inhibitor, 10 μM; Cl-amidine, a PAD-4 inhibitor, 200 μM). (**B**) The cell suspensions were spun onto a microscope slide by using a cytocentrifuge, fixed and subjected to immunofluorescence staining with an anti-MPO antibody (green), anti-histone antibody (red) and Hoechst (blue) nuclear stain. The supernatants were collected to measure the (**C**) IL-8 and (**D**) MPO levels using ELISA kits. (**E**) NET quantification as determined by immunofluorescence staining. Nine pictures from each group of experiments were randomly selected and analyzed by ImageJ. Bar: 25 μm. The averages of triplicate cultures ± SD are shown. Statistical significance was calculated using one-way ANOVA and Tukey’s post hoc test; *P* values were displayed, and for values less than 0.0001, they were represented as ****. Bar: 25 μm

To study the signaling pathway involved in CR Ab-induced NETosis, we used a range of inhibitors that target pathways that are related to neutrophil activation. These inhibitors included dasatinib, which acts as a Src family kinase (SFK) inhibitor; compound C, an AMP-activated protein kinase (AMPK) inhibitor; Ly294002, a phosphoinositide 3-kinase (PI3K) inhibitor; and Cl-amidine, a peptidylarginine deiminase 4 (PAD-4) inhibitor (Fig. 5A). These inhibitors did not cause any cytotoxicity in neutrophils at the doses we used, as determined by LDH release (data not shown). The effects of these inhibitors on preventing NETosis induced by mAb 127 and the RBD are shown in Fig. 5B. The results showed that inhibition of SFK signaling, which is a common factor that is upstream of both the ROS and PI3K pathways, with dasatinib effectively inhibited mAb 127- and RBD-triggered IL-8 production and NETosis. On the other hand, Ly294002 and Cl-amidine alleviated NET formation without affecting IL-8 secretion, while compound C suppressed IL-8 secretion but did not have a significant impact on NET formation (Fig. 5C-E). Given the observed relationship between NET-driven thrombosis and autoantibody production in patients with severe COVID-19, we further examined whether mAb 127 (in the presence of RBD) could trigger microthrombi formation in vitro. To achieve this, we established an in vitro thrombosis model by coculturing isolated neutrophils and platelets from healthy donors with HUVECs under various conditions. The results illustrated that microthrombi formation was induced only in the group that was treated with mAb 127 and the RBD in this coculture model (Fig. S8). Of note, the FDA-approved SFK inhibitor dasatinib exerted a significant inhibitory effect on the NETosis and thrombosis induced by mAb 127 and the RBD. Taken together, these results suggested that costimulation with mAb 127 and the RBD induced NETosis and thrombosis primarily through the SFK/PI3K/PAD4 signaling pathway in neutrophils.

### ACE2-cross-reactive anti-RBD antibodies in COVID-19 patient sera induce IL-8 secretion and NET formation in human neutrophils in the presence of RBD

**Fig. 6:**
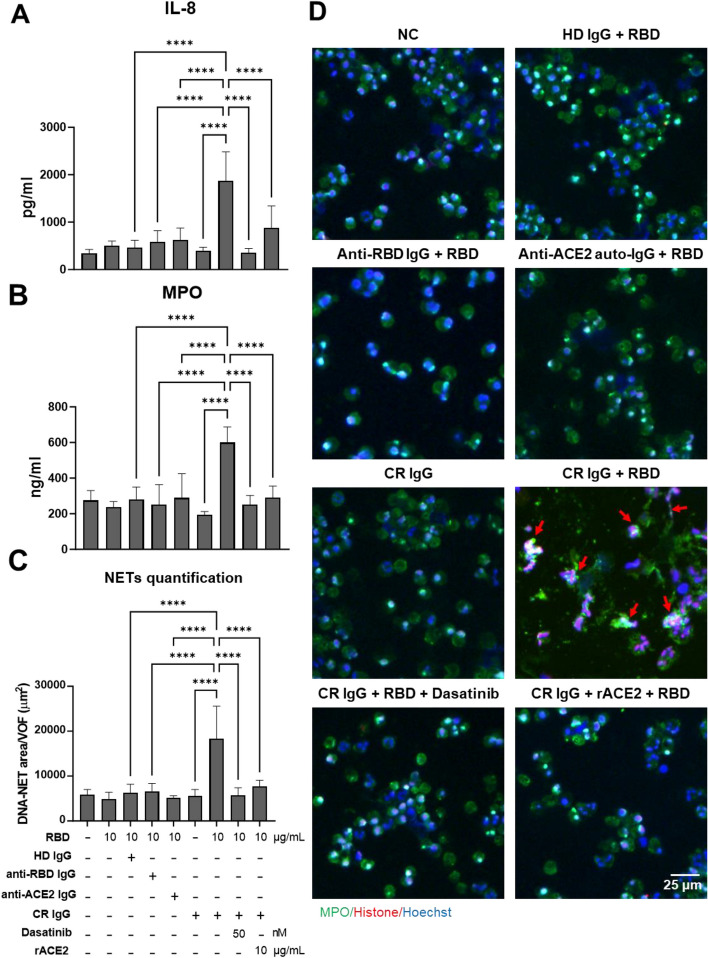
CR IgG purified from unvaccinated COVID-19 patients induces neutrophil activation and NETosis in the presence of RBD. Isolated human neutrophils were preincubated with 100 μg/mL purified IgG from HD serum (HD IgG) or different COVID-19 patient sera: anti-RBD IgG (anti-ACE2 antibody negative but anti-RBD antibody positive), anti-ACE2 IgG (anti-ACE2 antibody positive), or CR IgG (CR antibody positive) in the presence or absence of additional rACE2 (10 μg/mL) or dasatinib (50 nM) as indicated for 30 min. After removing unbound antibodies by centrifugation, cells were treated with the RBD protein (10 μg/mL) for 24 h. (**A**) IL-8 and (**B**) MPO levels in the supernatants were measured using ELISA kits. (**C**, **D**) Cell suspensions were cytocentrifuged onto microscope slides, fixed, and subjected to immunofluorescence staining with anti-MPO antibody (green), anti-histone antibody (red), and Hoechst nuclear stain (blue). The red arrow indicates neutrophils that released NETs. The area of NET formation was quantified using ImageJ. Averages of triplicate cultures ± SD are shown. Views for NET quantification were randomly selected with 9 pictures from each experiment. Statistical significance was assessed using one-way ANOVA and Tukey’s post hoc test; ****p < 0.0001. Bar: 25 μm

To validate that CR Abs in COVID-19 patient sera could trigger neutrophil activation or NET formation via the same mechanism as mAb127, we selected the serum sample from different groups based on the serological characteristics. The groups included healthy donor (HD) serum (anti-RBD negative, anti-ACE2 negative), anti-RBD serum (anti-RBD positive, anti-ACE2 negative), anti-ACE2 serum (anti-RBD positive, anti-ACE2 positive, CR antibody negative), and anti-CR serum (anti-RBD positive, anti-ACE2 positive, CR antibody positive). We then purified human total IgG from these sera using protein G agarose, ensuring that the purified total IgG maintained serological characteristics within a range of 100–200 μg/mL (data not shown). Isolated human neutrophils were preincubated with 100 μg/mL purified IgG from different serum, in the presence or absence of additional rACE2. Consistent with the results observed with mAb 127 stimulation, in the presence of RBD, neutrophil activation (including IL-8, MPO, and NETosis) was observed specifically in the purified CR IgG group, but not in the purified anti-ACE2 IgG or purified anti-RBD IgG groups (Fig. 6). These results indicate that the observed effect is unique to CR Abs. Importantly, the neutrophil activation caused by purified CR IgG could be effectively inhibited by ACE2 and dasatinib (Fig. 6). Crucially, these CR Abs retained their pathological function in COVID-19 patient sera (Fig. S9). IL-8 and MPO secretion as well as NET formation were observed only in the CR-Ab-containing sera groups in the presence of RBD but not in the other groups (Fig. S9). Furthermore, CR serum-triggered neutrophil activation and NETosis could be blocked by incubation with recombinant human ACE2 or the Src inhibitor dasatinib (Fig. S9). Taken together, these results further suggest that the presence of CR Abs, but not anti-ACE2 autoAbs, contributes to the enhancement of the inflammatory response and NETosis in COVID-19.

### Vaccination prevents the production of ACE2-cross-reactive anti-RBD antibodies in COVID-19

**Fig. 7:**
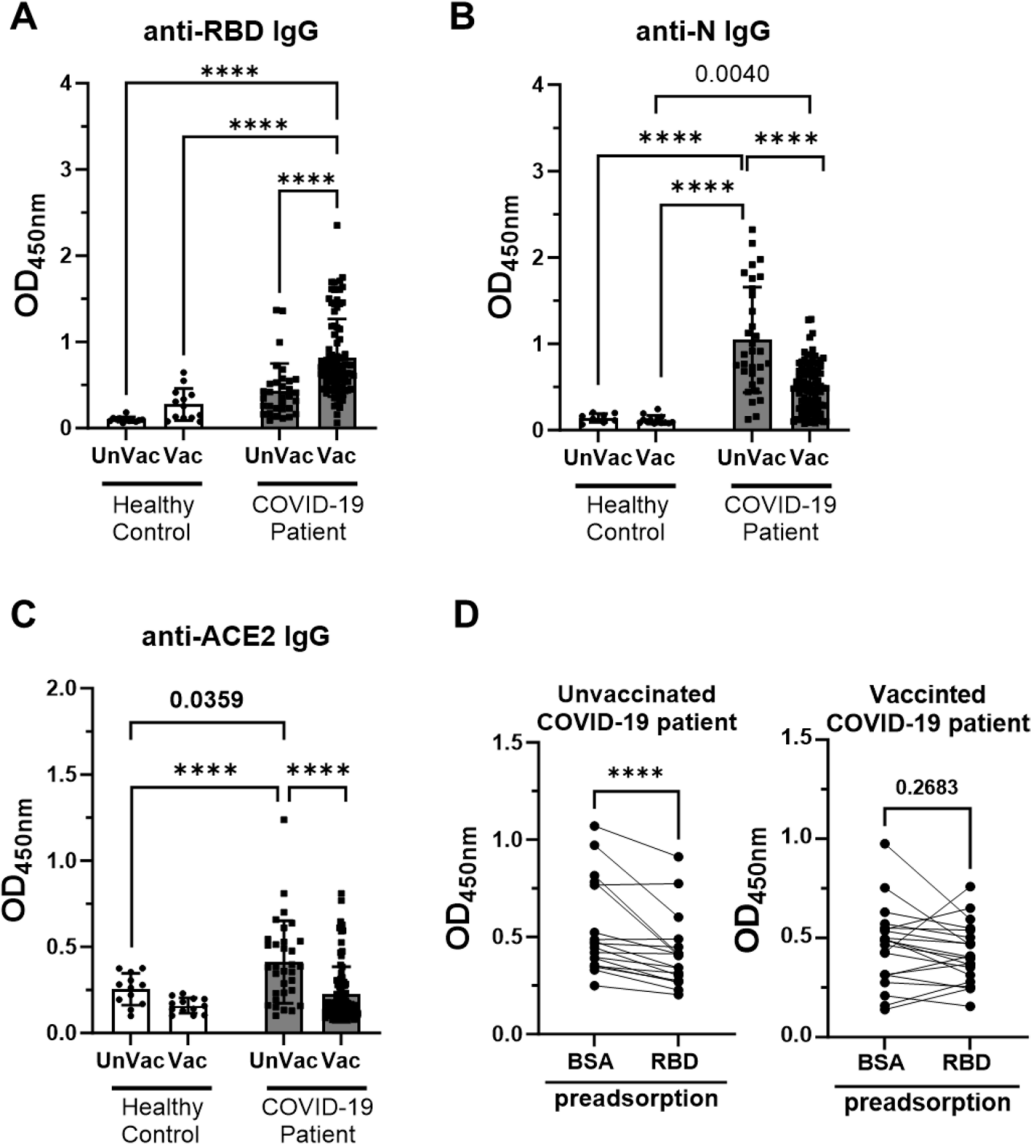
Vaccination is conducive to decreasing CR Abs IgG levels in the sera of COVID-19 patients. IgG against (**A**) RBD, (**B**) nucleocapsid, N and (**C**) ACE2 proteins in sera from different groups were measured by ELISA. (**D**) Anti-ACE2 IgG levels in sera of unvaccinated COVID-19 patients and vaccinated COVID-19 patients after BSA or RBD preadsorption. Multiple comparisons in COVID-19 samples were conducted by two-way ANOVA. The comparison between BSA and RBD preadsorption was analyzed by paired Student’s t-test; *P* values were displayed, and for values less than 0.0001, they were represented as ****

To investigate the contribution of vaccination to CR Ab production in COVID-19, we compared serum samples from various groups of individuals with or without prior immunization with COVID-19 AZ or mRNA vaccines before SARS-CoV-2 infection (Supplementary Table 1). A significant elevation in anti-RBD IgG levels was observed in vaccinated COVID-19 patients compared to those in healthy cohorts and unvaccinated COVID-19 patients (Fig. 7A). On the other hand, the levels of IgG against SARS-CoV-2 nucleocapsid (N) were notably higher in sera from unvaccinated COVID-19 patients than in those from healthy cohorts and vaccinated COVID-19 patients (Fig. 7B). Moreover, IgG levels against human ACE2 were particularly elevated in unvaccinated COVID-19 patients with severe disease (Fig. 7C). Subsequently, we performed the preadsorption test to confirm whether these anti-ACE2 IgG antibodies are CR Abs or simply anti-ACE2 autoantibodies. The results showed a significant decrease in ACE2-binding IgG levels from unvaccinated COVID-19 patients after RBD preadsorption, but similar results were not observed in the vaccinated COVID-19 patient group, indicating a significant increase in CR Ab levels in the unvaccinated COVID-19 patient group (Fig. 7D). In fact, we found that not only disease severity but also the positive rate of CR Abs was significantly decreased in vaccinated COVID-19 patients compared to the unvaccinated COVID-19 patient group (Table 1).

**Table 1:** Distribution of disease severity, anti-ACE2 autoantibody and CR antibody positive rate in COVID-19 patients with vaccination (Vac) or without (UnVac)

**Demographic**	**UnVac (*n* = 31)**	**Vac (*n* = 89)**	***P* value**^a^
Disease severity			< 0.0001
Mild	4 (12.9%)	63 (70.8%)	
Positive for Anti-ACE2 autoAb	1 (25%)	7 (11.1%)	ns
Positive for CR Ab	0	1 (1.6%)	ns
Moderate/Severe	27 (87.1%)	26 (29.2%)	
Positive for Anti-ACE2 autoAb	18 (66.7%)	7 (26.9%)	0.058
Positive for CR Ab	9 (33.3%)	1 (3.8%)	0.011

*Abbreviation:* *ns* not significant

^a^The Fisher exact test was used to determine the *P* values

## Discussion

**Fig. 8:**
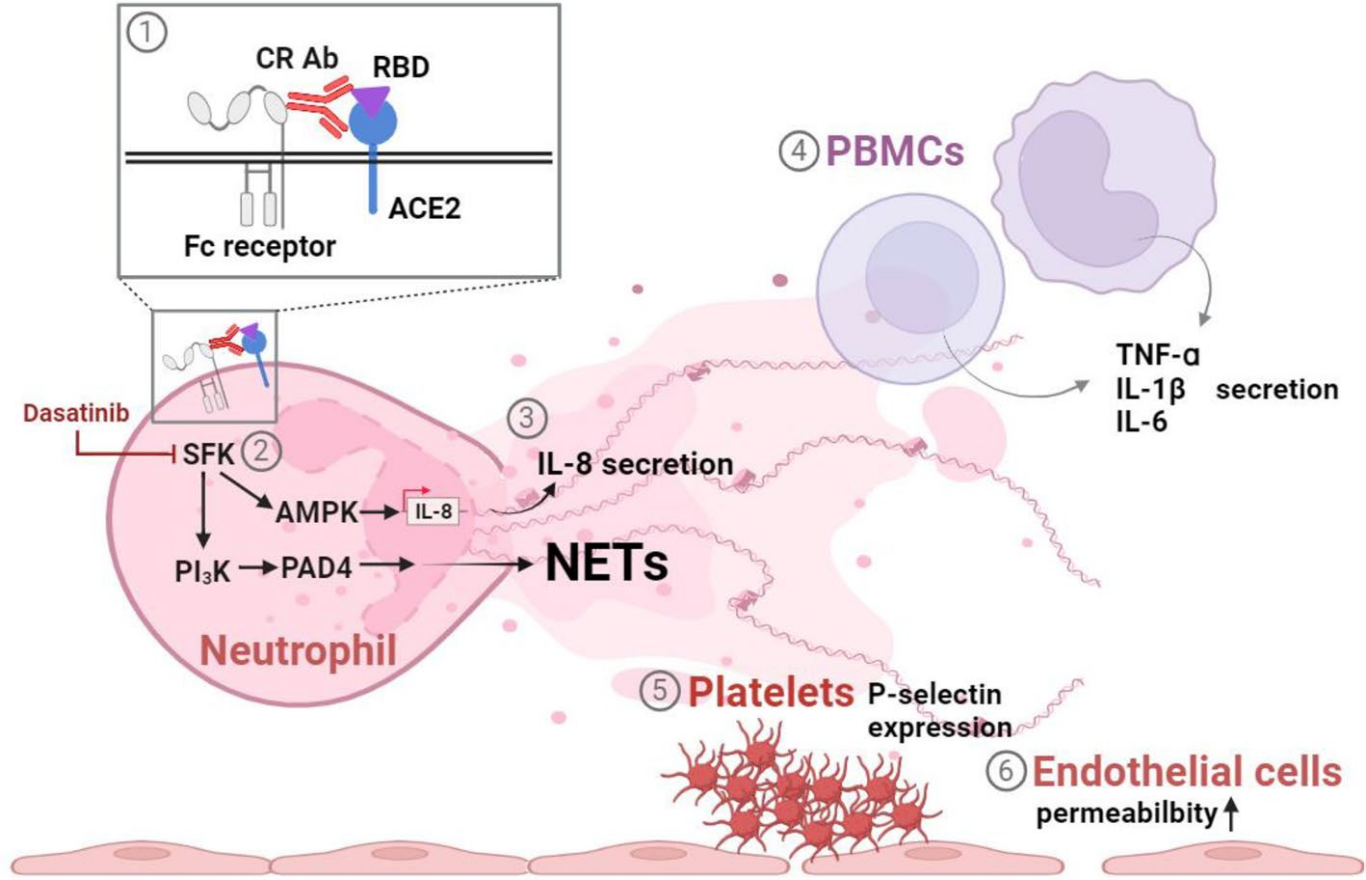
The potential mechanisms underlying CR Ab-induced thrombosis. (1) CR Abs binding to RBD and ACE2 as well as Fc receptors on the surface of neutrophils induce signaling pathways. (2) Signaling pathways that regulate IL-8 secretion and NET formation are activated: SFK-AMPK signaling mediates IL-8 secretion, while SFK-PI3K-PAD4 signaling mediates NET formation. The actions of both IL-8 and NETs could be suppressed by dasatinib, which is an SFK inhibitor. (3) Secreted IL-8 and NETs subsequently cause (4) the secretion of TNFα, IL-6, and IL-1β from PBMCs, (5) enhanced expression of surface P-selectin on platelets, and (6) increased endothelial permeability. These processes collectively contribute to the eventual formation of thrombi. CR Ab, ACE2-cross-reactive RBD antibodies; ACE2, angiotensin converting enzyme-2; RBD, SARS-CoV-2 receptor binding domain; IL-8, interleukin-8; NETs, neutrophil extracellular traps; SFK, Src family kinases; AMPK, AMP-activated protein kinase; PI3K, phosphoinositide 3-kinase; PAD4, protein arginine deiminase 4; TNFα, tumor necrosis factor alpha; IL-6, interleukin-6; IL-1β, interleukin-1β; PBMC, peripheral blood mononuclear cells. Created with BioRender.com

It has been reported that NET formation and autoantibody production in patients with severe COVID-19 may contribute to high-profile thrombus events and organ damage [14, 16, 25, 26]. However, the relationship between these factors and their role in immunothrombosis during COVID-19 remains unclear. In our previous study, we identified ACE2-cross-reactive anti-RBD antibodies (CR Abs) as one of the sources of anti-ACE2 autoantibodies in both COVID-19 patients and RBD-immunized mice [20]. In this study, we revealed that CR Ab levels were significantly increased in the sera of unvaccinated COVID-19 patients compared to vaccinated COVID-19 patients. In addition, CR Ab levels were also higher in patients with moderate/severe COVID-19 than in patients with mild COVID-19. These results suggest that CR Abs may play a role in the severity of COVID-19, and vaccination may reduce the production of CR Abs. Furthermore, we demonstrated that CR Abs present in the sera of COVID-19 patients could activate neutrophils and induce NETosis when RBD was present. These effects were similar to the effects of mAb 127, a monoclonal CR Abs derived from RBD-immunized mice, on neutrophils, which could be blocked by ACE2 or dasatinib. Based on our in vitro study results, the potential mechanisms by which CR Abs induce NETosis and thrombosis are summarized in Fig. 8. However, the contribution of CR Abs to NET formation and thrombosis in COVID-19 patients needs further investigation in vivo.

Anti-ACE2 autoantibodies have been recognized as a marker of disease severity and have been associated with proinflammatory responses in COVID-19 [18]. Some studies pointed out that the participation of anti-ACE2 in the pro-inflammatory response during COVID-19 is linked to its inhibitory effect on the enzymatic activity of ACE2, leading to an increase in angiotensin II levels that promote inflammation [17]. Other studies have indicated that anti-ACE2 IgM from COVID-19 patients’ sera activated complement and led to vasculopathy in vitro [19]. Moreover, even anti-ACE2 antibodies lacking blocking activity have been proposed to amplify the pathogenic inflammatory response [27]. Similarly, our results showed that anti-ACE2 was highly increased in severe patients with COVID-19 along with CR Abs. Interestingly, the CR Ab (mAb 127) we used in this study can cause complement-dependent cytotoxicity in ACE2-overexpressing HEK293 cells, although it did not inhibit the enzymatic activity of recombinant ACE2 in vitro (data not shown). Nevertheless, our studies on CR Abs-enhanced NETosis provided another possible mechanism of anti-ACE2-correlated inflammatory response in COVID-19.

Exacerbated NET formation is one of the main causes of multiorgan damage and acute complications and leads to clinical deterioration of symptoms and mortality in COVID-19 [28, 29]. However, the mechanisms that may contribute to COVID-19-induced NETosis are very complex. Veras et al. reported that SARS-CoV-2 replication/infection in human neutrophils is necessary for triggering the release of NETs, as inactivated SARS-CoV-2 failed to induce NET release [12]. On the other hand, two other studies demonstrated that the SARS-CoV-2 spike protein can directly induce neutrophil degranulation and NET formation by stimulating human neutrophils with recombinant spike protein or spike-expressing lentivirus [30, 31]. Platelets have also been identified as a key factor in NET formation in COVID-19 [32, 33]. Furthermore, several studies suggested that immunocomplex (or spike-specific IgA/IgG immune complexes) can trigger NET formation and correlate to severe COVID-19 [34–36]. In this study, we provide a different mechanistic explanation for NET formation during acute COVID-19 infection by demonstrating that CR Abs may also contribute to NET formation in COVID-19. Importantly, we found that co-stimulation with CR Abs and RBD triggered NETosis via the SFK/PI3K/PAD4 signaling pathway, and our results suggest that blocking neutrophil activation by ACE2 or dasatinib may be a potential therapeutic strategy to prevent NETosis-induced immunothrombosis in COVID-19.

In addition to acute SARS-CoV-2 infection, multiple studies have suggested that thrombosis-promoting autoantibodies and NET formation contribute to the pathology of long COVID-19, which refers to the persistence of COVID-19 symptoms beyond the acute phase [37]. Long COVID-19 is estimated to affect at least 10% of COVID-19 patients, and it has been associated with the continued presence of the spike protein in the bloodstream even one year after infection [38]. Clinical reports have also noted elevated levels of autoantibodies, including anti-ACE2 autoantibodies, in long COVID patients directly related to neutrophil hyperactivation [39]. Importantly, a recent cohort study showed that NETosis was maintained at a higher level in post-COVID syndrome with increased coagulation markers to exacerbate disease progression [40]. Here, we revealed that when CR Abs and the RBD protein (in the form of shedding spikes) coexist in the bloodstream, NETosis can be continually induced, irrespective of whether the virus has been cleared. This finding provides a potential explanation and identifies therapeutic targets for autoantibody-triggered NETosis in the development of long COVID-19.

Numerous cohort studies have reported that COVID-19 vaccination is highly effective in preventing SARS-CoV-2 infection, reducing COVID-19 mortality rates, and lowering the occurrence of long COVID-19 [41–43]. Consistently, our results suggest an illustration of vaccination for the prevention of severe COVID-19 by diminishing the production of CR Abs. Several mechanisms for the benefit of vaccination have been suggested, including avoidance of dysregulation in the B-cell response and suppression of the influence of pathological inflammation [44–46]. It has been demonstrated that excessive extrafollicular B‐cell responses with lower somatic hypermutation (SHM) are favored in SARS-CoV-2 infection. In contrast, the vaccine boosted B cells to undergo a germinal center response with higher SHM [46]. These studies align with our observation that CR Abs were increased in unvaccinated severe patients with COVID-19. Taken together, our findings suggest a potential pathogenic role of CR Abs in exacerbating COVID-19 by promoting NETosis, highlighting ACE2 and dasatinib as potential treatments and emphasizing the importance of vaccination in reducing CR Abs production.

## Conclusions

In this study, we reveal that CR Abs in the sera of COVID-19 patients might exacerbate COVID-19 disease severity by promoting NETosis through interacting with ACE2 and the Fc receptor. Recombinant ACE2 or dasatinib shows potential in rescuing CR Abs-induced NETosis and immunothrombosis. Additionally, COVID-19 vaccination could prevent CR Abs production and reduce disease severity during SARS-CoV-2 infection. These findings offer insights into potential therapeutic interventions and preventive strategies, contributing to a deeper understanding of COVID-19 pathogenesis and suggesting ways to mitigate its severity.

### Supplementary Information

**Additional file 1.**

## Data Availability

The datasets used and/or analyzed during the current study are available from the first authors on reasonable request.

## References

[1] Al-Samkari H, Karp Leaf RS, Dzik WH, Carlson JCT, Fogerty AE, Waheed A (2020). COVID-19 and coagulation: bleeding and thrombotic manifestations of SARS-CoV-2 infection. Blood.

[2] Shang J, Ye G, Shi K, Wan Y, Luo C, Aihara H (2020). Structural basis of receptor recognition by SARS-CoV-2. Nature.

[3] Al-Sheboul SA, Brown B, Shboul Y, Fricke I, Imarogbe C, Alzoubi KH (2022). An Immunological review of SARS-CoV-2 infection and vaccine serology: innate and adaptive responses to mRNA, adenovirus, inactivated and protein subunit vaccines. Vaccines (Basel).

[4] Antonelli M, Penfold RS, Merino J, Sudre CH, Molteni E, Berry S (2022). Risk factors and disease profile of post-vaccination SARS-CoV-2 infection in UK users of the COVID Symptom Study app: a prospective, community-based, nested, case-control study. Lancet Infect Dis.

[5] Lamers MM, Haagmans BL (2022). SARS-CoV-2 pathogenesis. Nat Rev Microbiol.

[6] Ramos-Casals M, Brito-Zeron P, Mariette X (2021). Systemic and organ-specific immune-related manifestations of COVID-19. Nat Rev Rheumatol.

[7] Zuo Y, Yalavarthi S, Shi H, Gockman K, Zuo M, Madison JA (2020). Neutrophil extracellular traps in COVID-19. JCI Insight.

[8] Liana P, Liberty IA, Murti K, Hafy Z, Salim EM, Zulkarnain M, Umar TP (2022). A systematic review on neutrophil extracellular traps and its prognostication role in COVID-19 patients. Immunol Res.

[9] Liu X, Arfman T, Wichapong K, Reutelingsperger CPM, Voorberg J, Nicolaes GAF (2021). PAD4 takes charge during neutrophil activation: Impact of PAD4 mediated NET formation on immune-mediated disease. J Thromb Haemost.

[10] Papayannopoulos V (2018). Neutrophil extracellular traps in immunity and disease. Nat Rev Immunol.

[11] Ackermann M, Anders HJ, Bilyy R, Bowlin GL, Daniel C, De Lorenzo R (2021). Patients with COVID-19: in the dark-NETs of neutrophils. Cell Death Differ.

[12] Veras FP, Pontelli MC, Silva CM, Toller-Kawahisa JE, de Lima M, Nascimento DC (2020). SARS-CoV-2-triggered neutrophil extracellular traps mediate COVID-19 pathology. J Exp Med.

[13] Zhu Y, Chen X, Liu X (2022). NETosis and neutrophil extracellular traps in COVID-19: immunothrombosis and beyond. Front Immunol.

[14] Middleton EA, He XY, Denorme F, Campbell RA, Ng D, Salvatore SP (2020). Neutrophil extracellular traps contribute to immunothrombosis in COVID-19 acute respiratory distress syndrome. Blood.

[15] Wang EY, Mao T, Klein J, Dai Y, Huck JD, Jaycox JR (2021). Diverse functional autoantibodies in patients with COVID-19. Nature.

[16] Cabral-Marques O, Halpert G, Schimke LF, Ostrinski Y, Vojdani A, Baiocchi GC (2022). Autoantibodies targeting GPCRs and RAS-related molecules associate with COVID-19 severity. Nat Commun.

[17] Arthur JM, Forrest JC, Boehme KW, Kennedy JL, Owens S, Herzog C (2021). Development of ACE2 autoantibodies after SARS-CoV-2 infection. Plos One.

[18] Rodriguez-Perez AI, Labandeira CM, Pedrosa MA, Valenzuela R, Suarez-Quintanilla JA, Cortes-Ayaso M (2021). Autoantibodies against ACE2 and angiotensin type-1 receptors increase severity of COVID-19. J Autoimmun.

[19] Casciola-Rosen L, Thiemann DR, Andrade F, Trejo-Zambrano MI, Leonard EK, Spangler JB (2022). IgM anti-ACE2 autoantibodies in severe COVID-19 activate complement and perturb vascular endothelial function. JCI Insight.

[20] Lai YC, Cheng YW, Chao CH, Chang YY, Chen CD, Tsai WJ (2022). Antigenic cross-reactivity between SARS-CoV-2 S1-RBD and its receptor ACE2. Front Immunol.

[21] Cheng YL, Chao CH, Lai YC, Hsieh KH, Wang JR, Wan SW (2022). Antibodies against the SARS-CoV-2 S1-RBD cross-react with dengue virus and hinder dengue pathogenesis. Front Immunol.

[22] Wilhelmsen K, Farrar K, Hellman J. Quantitative in vitro assay to measure neutrophil adhesion to activated primary human microvascular endothelial cells under static conditions. J Vis Exp. 2013;78:e50677. 10.3791/50677 PMC3856291 23995778

[23] Tong M, Abrahams VM (2021). Visualization and quantification of neutrophil extracellular traps. Methods Mol Biol.

[24] Najmeh S, Cools-Lartigue J, Giannias B, Spicer J, Ferri LE (2015). Simplified human neutrophil extracellular traps (NETs) isolation and handling. J Vis Exp.

[25] Szturmowicz M, Demkow U (2021). Neutrophil extracellular traps (NETs) in severe SARS-CoV-2 lung disease. Int J Mol Sci.

[26] Radermecker C, Detrembleur N, Guiot J, Cavalier E, Henket M, d'Emal C (2020). Neutrophil extracellular traps infiltrate the lung airway, interstitial, and vascular compartments in severe COVID-19. J Exp Med.

[27] Chang SE, Feng A, Meng W, Apostolidis SA, Mack E, Artandi M (2021). New-onset IgG autoantibodies in hospitalized patients with COVID-19. Nat Commun.

[28] Perico L, Benigni A, Casiraghi F, Ng LFP, Renia L, Remuzzi G (2021). Immunity, endothelial injury and complement-induced coagulopathy in COVID-19. Nat Rev Nephrol.

[29] Jamal M, Bangash HI, Habiba M, Lei Y, Xie T, Sun J (2021). Immune dysregulation and system pathology in COVID-19. Virulence.

[30] Youn YJ, Lee YB, Kim SH, Jin HK, Bae JS, Hong CW (2021). Nucleocapsid and spike proteins of SARS-CoV-2 drive neutrophil extracellular trap formation. Immune Netw.

[31] Lebourgeois S, David A, Chenane HR, Granger V, Menidjel R, Fidouh N (2022). Differential activation of human neutrophils by SARS-CoV-2 variants of concern. Front Immunol.

[32] Skendros P, Mitsios A, Chrysanthopoulou A, Mastellos DC, Metallidis S, Rafailidis P (2020). Complement and tissue factor-enriched neutrophil extracellular traps are key drivers in COVID-19 immunothrombosis. J Clin Invest.

[33] Sung PS, Yang SP, Peng YC, Sun CP, Tao MH, Hsieh SL (2022). CLEC5A and TLR2 are critical in SARS-CoV-2-induced NET formation and lung inflammation. J Biomed Sci.

[34] Mazzitelli I, Bleichmar L, Luduena MG, Pisarevsky A, Labato M, Chiaradia V (2021). Immunoglobulin G immune complexes may contribute to neutrophil activation in the course of severe coronavirus disease 2019. J Infect Dis.

[35] Ankerhold J, Giese S, Kolb P, Maul-Pavicic A, Voll RE, Göppert N (2022). Circulating multimeric immune complexes contribute to immunopathology in COVID-19. Nat Commun.

[36] Allen KC, Ramos-Benitez MJ, Teague H, Chertow DS, Strich JR, Warner S (2023). 249 SARS-CoV-2 spike immune complexes induce NETosis in COVID-19. Open Forum Infect Dis.

[37] Turner S, Khan MA, Putrino D, Woodcock A, Kell DB, Pretorius E (2023). Long COVID: pathophysiological factors and abnormalities of coagulation. Trends Endocrinol Metab.

[38] Lechuga GC, Morel CM, De-Simone SG (2023). Hematological alterations associated with long COVID-19. Front Physiol.

[39] Chen C, Amelia A, Ashdown GW, Mueller I, Coussens AK, Eriksson EM (2021). Risk surveillance and mitigation: autoantibodies as triggers and inhibitors of severe reactions to SARS-CoV-2 infection. Mol Med.

[40] Krinsky N, Sizikov S, Nissim S, Dror A, Sas A, Prinz H (2023). NETosis induction reflects COVID-19 severity and long COVID: insights from a 2-center patient cohort study in Israel. J Thromb Haemost.

[41] Brannock MD, Chew RF, Preiss AJ, Hadley EC, Redfield S, McMurry JA (2023). Long COVID risk and pre-COVID vaccination in an EHR-based cohort study from the RECOVER program. Nat Commun.

[42] Peng K, Li X, Yang D, Chan SCW, Zhou J, Wan EYF (2023). Risk of autoimmune diseases following COVID-19 and the potential protective effect from vaccination: a population-based cohort study. EClinicalMedicine.

[43] Byambasuren O, Stehlik P, Clark J, Alcorn K, Glasziou P (2023). Effect of covid-19 vaccination on long covid: systematic review. BMJ Med.

[44] Jaycox JR, Lucas C, Yildirim I, Dai Y, Wang EY, Monteiro V (2023). SARS-CoV-2 mRNA vaccines decouple anti-viral immunity from humoral autoimmunity. Nat Commun.

[45] Cao T, Liu L, To KK, Lim CY, Zhou R, Ming Y (2022). Mitochondrial regulation of acute extrafollicular B-cell responses to COVID-19 severity. Clin Transl Med.

[46] Roltgen K, Boyd SD (2021). Antibody and B cell responses to SARS-CoV-2 infection and vaccination. Cell Host Microbe.

