# Supplementary material for: Enhancement of NETosis by ACE2-cross-reactive anti-SARS-CoV-2 RBD antibodies in patients with COVID-19
Source: J Biomed Sci. 2024 Apr 18;31:39. doi: 10.1186/s12929-024-01026-5 (PMC11027296; doi:10.1186/s12929-024-01026-5)
Supplement: Supplementary file 1 — Additional file 1. [file 12929_2024_1026_MOESM1_ESM.docx]

**SUPPLEMENTARY DATA**


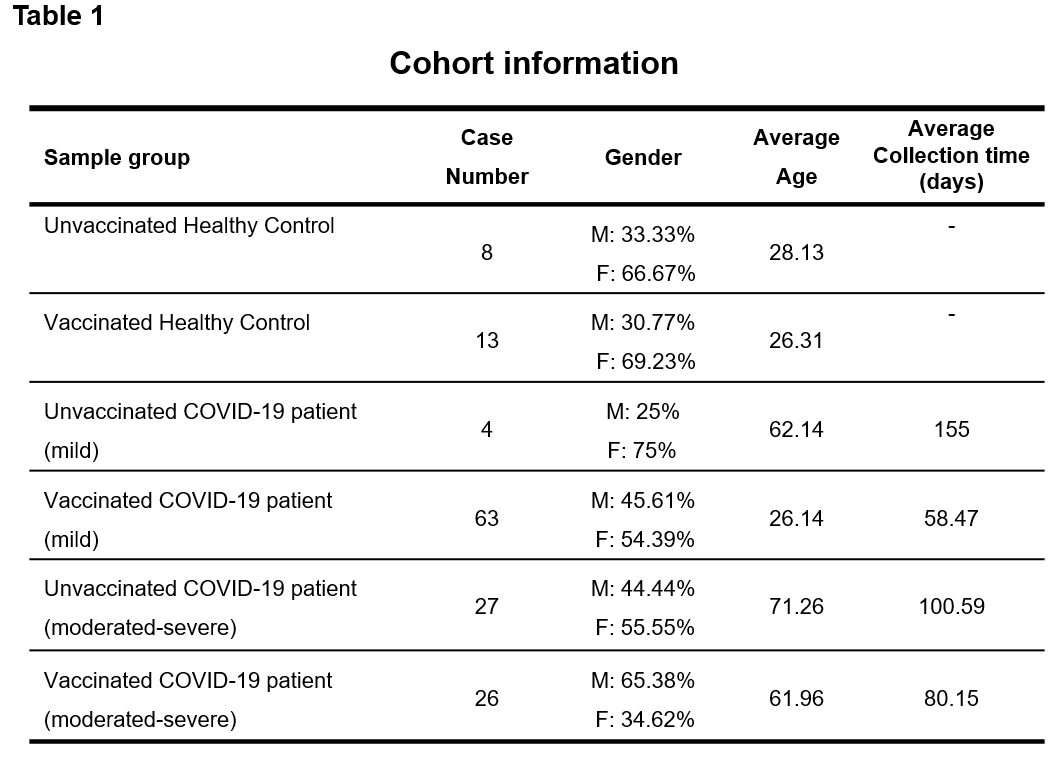


**TABLE 1 Demographic characteristics of COVID-19 patients and healthy controls who were or were not vaccinated.** The definition of mild illness: individuals who had any of the various signs and symptoms of COVID-19 (e.g., fever, cough, sore throat, malaise, headache, muscle pain, nausea, vomiting, diarrhea, loss of taste and smell) but did not have shortness of breath, dyspnea, or abnormal chest imaging. The definition of moderate/severe illness was as follows: individuals who experienced a fever (≥38℃) or respiratory symptoms and subsequently developed pneumonia requiring oxygen therapy or other complications within 14 days (inclusive), leading to hospitalization (including emergency room admission). M, male; F, female.

**
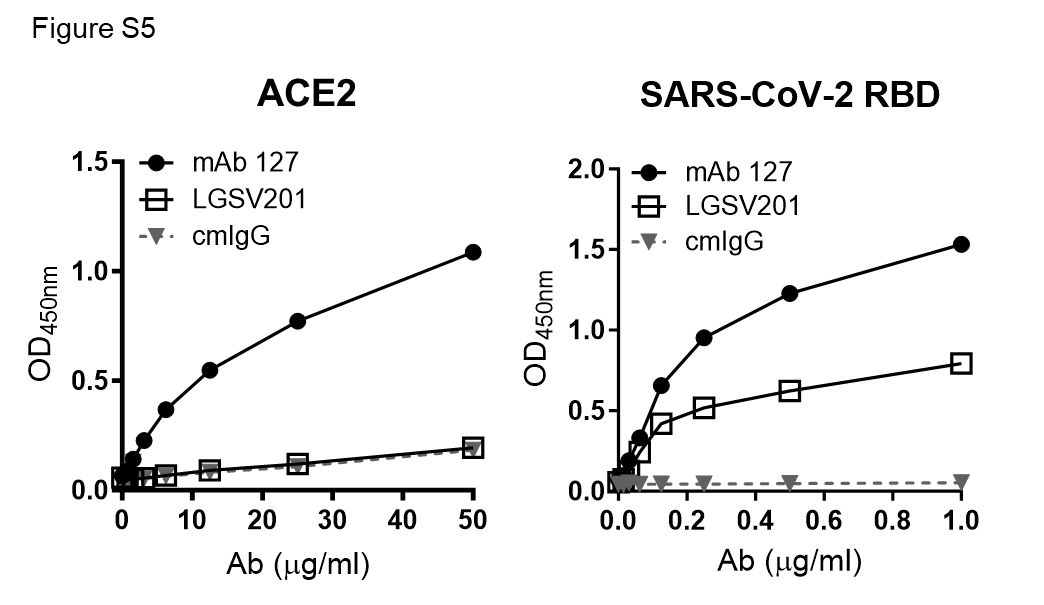
Fig. S1 Antigen binding properties of mAb 127** **and LGSV201.** Binding abilities of mAbs 127, LGSV201 and cmIgG to (A) human ACE2 and (B) the RBD as determined with ACE2- or RBD-coated ELISA plates.

**
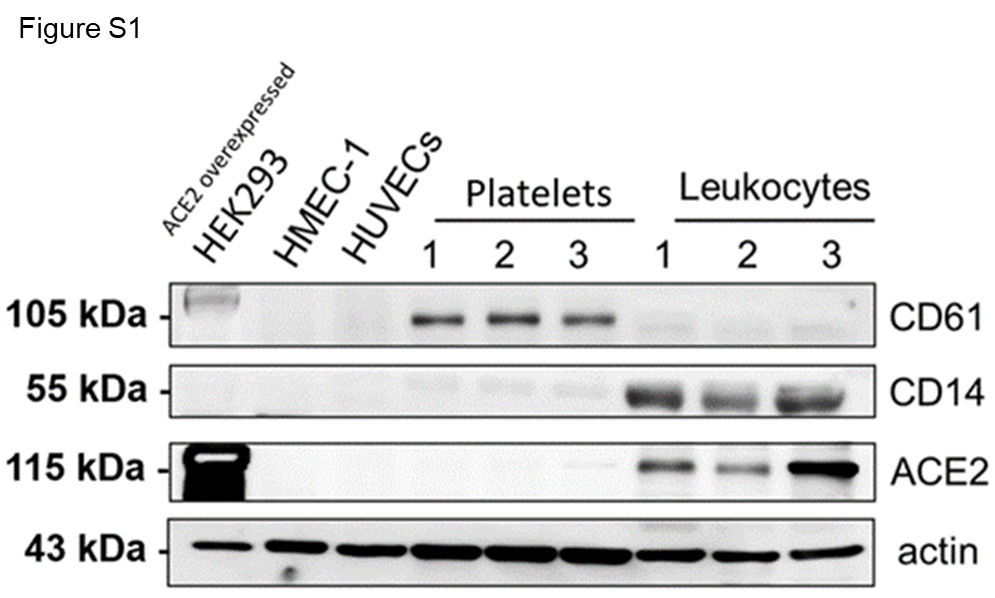
Fig. S2 The expression level of ACE2 in endothelial cells, platelets, and leukocytes.** Cell lysates of ACE2-overexpressing HEK293 cells, a human endothelial cell line (HMEC-1 cells), HUVECs, freshly isolated human platelets and leukocytes from three different healthy donors were separated using SDS‒PAGE, and the expression levels of CD61 (platelet marker), CD14 (PBMC marker), and ACE2 in each sample were measured by western blotting using specific antibodies (A12737, ABclonal, College Park, MD); sc-1182, Santa Cruz Biotechnology; GTX61848, Genetex).

**
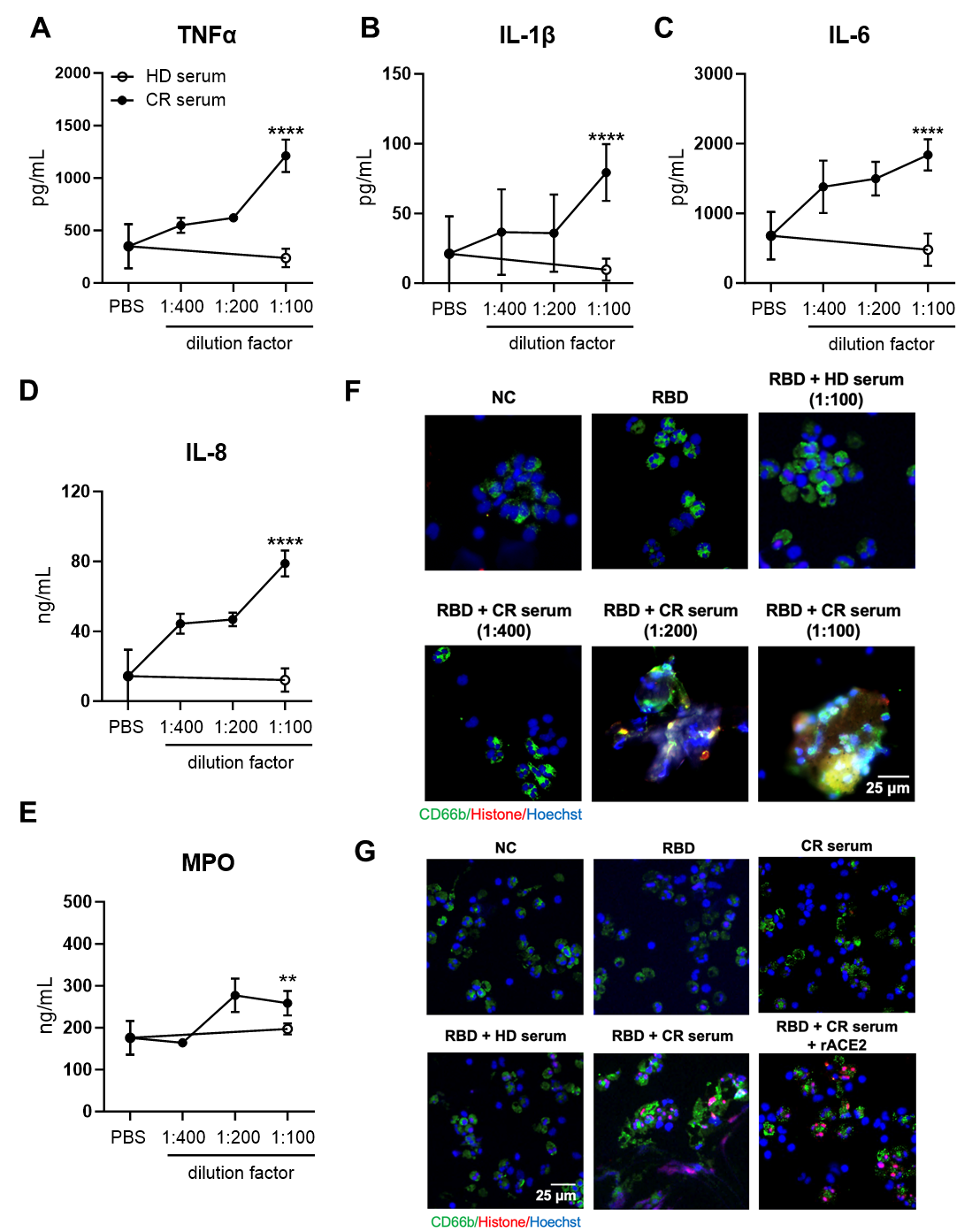
**

**Fig. S3 CR Abs from the sera of unvaccinated COVID-19 patients induced leukocyte cytokine secretion and NET formation in the presence of RBD.** Isolated human leukocytes were preincubated with different dilutions of healthy donor serum (HD serum) or CR Ab-containing COVID-19 patient sera (CR serum) as indicated for 30 min. Afterward, unbound antibodies were removed by centrifugation, and the cells were treated with the RBD protein (10 μg/mL) for 24 h. The supernatants were collected to measure the (A) TNF-α, (B) IL-1β, (C) IL-6, (D) IL-8 and (E) MPO levels using ELISA kits. (F) The cell suspensions were spun onto a microscope slide by using a cytocentrifuge and were fixed and subjected to immunofluorescence staining with an anti-CD66b antibody (green), anti-histone antibody (red) and Hoechst (blue) nuclear stain. In addition, the effect of rACE2 on CR serum (1:100 dilution) induced NETosis in the presence of RBD is shown in (G). **p < 0.01, ****p < 0.0001.

**
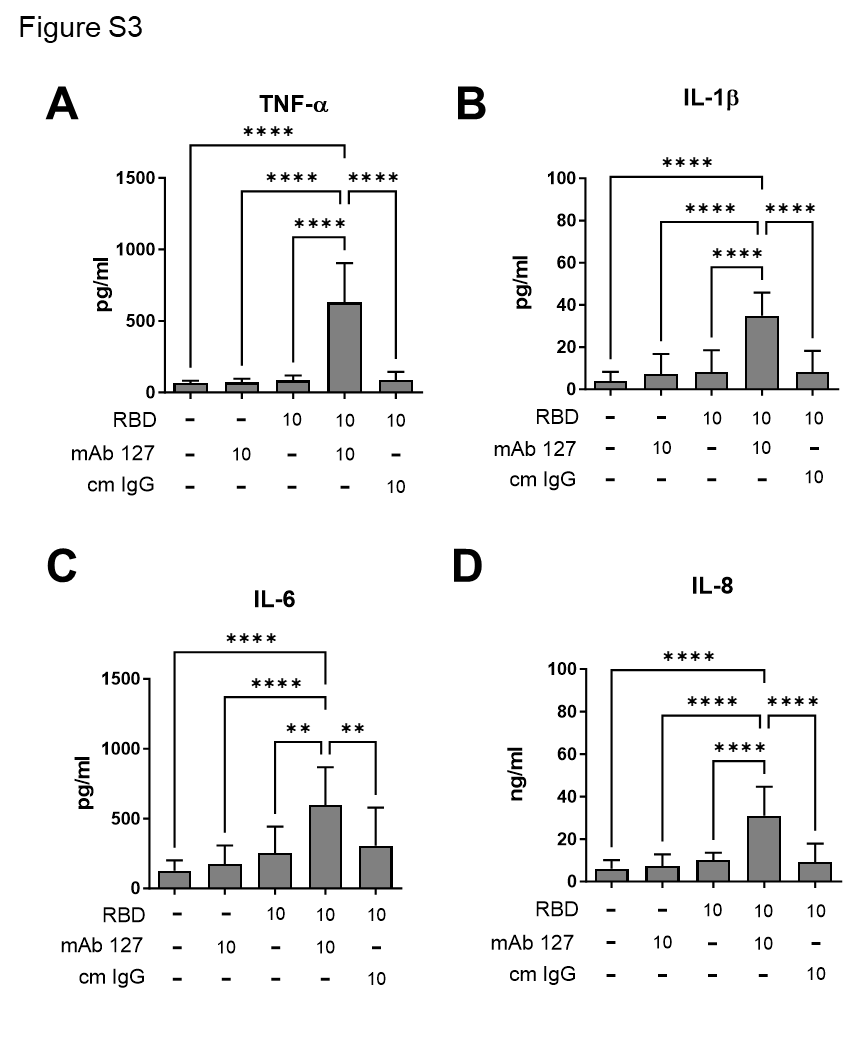
Fig. S4 Cytokine secretion by isolated human leukocytes incubated with CR Abs in the presence of RBD.** Isolated human leukocytes were treated with the recombinant RBD protein in the presence or absence of the indicated antibodies (mAb 127 or isotype control mouse IgG) for 24 h. The supernatants were collected to measure the (A) TNF-α, (B) IL-1β, (C) IL-6, and (D) IL-8 levels using ELISA kits. The averages of triplicate cultures ± SD are shown. Statistical significance was calculated using one-way ANOVA and Tukey’s post hoc test, **p < 0.01, ****p < 0.0001.


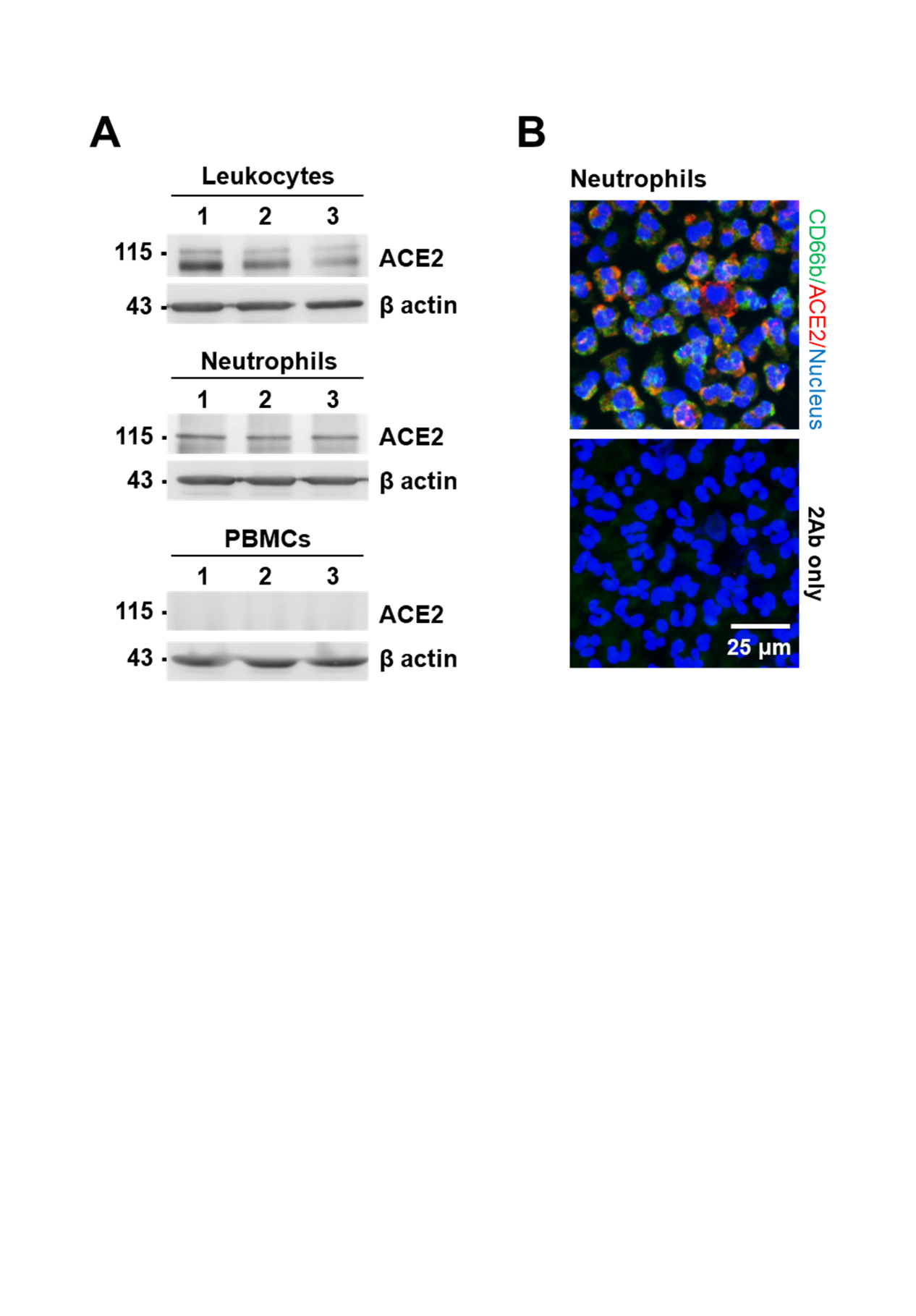
**Fig. S5 Human neutrophils but not PBMCs are the main type of cells that express ACE2 in leukocytes.** (A) ACE2 protein expression levels in isolated human leukocytes, neutrophils and PBMC lysates obtained from three different healthy donors were determined by western blotting using anti-ACE2 antibodies. CD14 was used as a marker of PBMCs, and β-actin was used as an internal control. (B) Isolated human neutrophils were spun onto a microscope slide by using a cytocentrifuge, fixed and stained with anti-CD66b antibodies (green), anti-ACE2 antibodies (red), and DAPI (blue) nuclear and then visualized using immunofluorescence microscopy (Olympus FluoView FV1000).


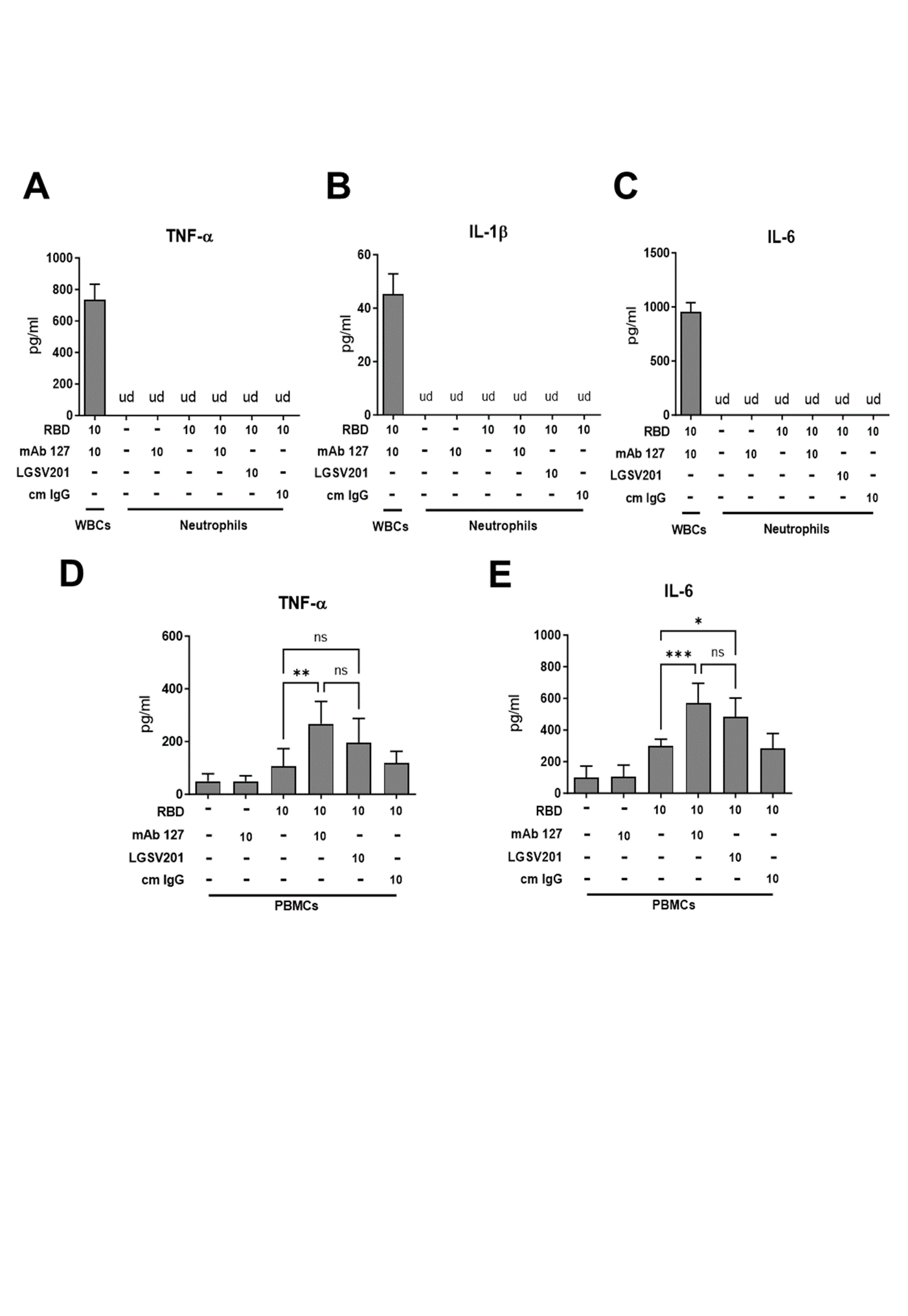
**Fig. S6 TNF-α and IL-6 secretion by PBMCs but not neutrophils is induced by both mAb 127 and LGSV201 in the presence of the RBD.** Isolated human leukocytes, as a positive control, neutrophils, or PBMCs were treated with the recombinant RBD protein in the presence or absence of the indicated antibodies (mAbs 127, LGSV201, or cmIgG) for 24 h. The levels of (A) TNF-α, (B) IL-1β, and (C) IL-6 in the neutrophil supernatants as well as the levels of (D) TNF-α and (E) IL-6 in the PBMC supernatants after stimulation were measured using ELISA kits. The averages of triplicate cultures ± SD are shown. *p < 0.05, **p < 0.01, ***p < 0.001, ns: non-significant.


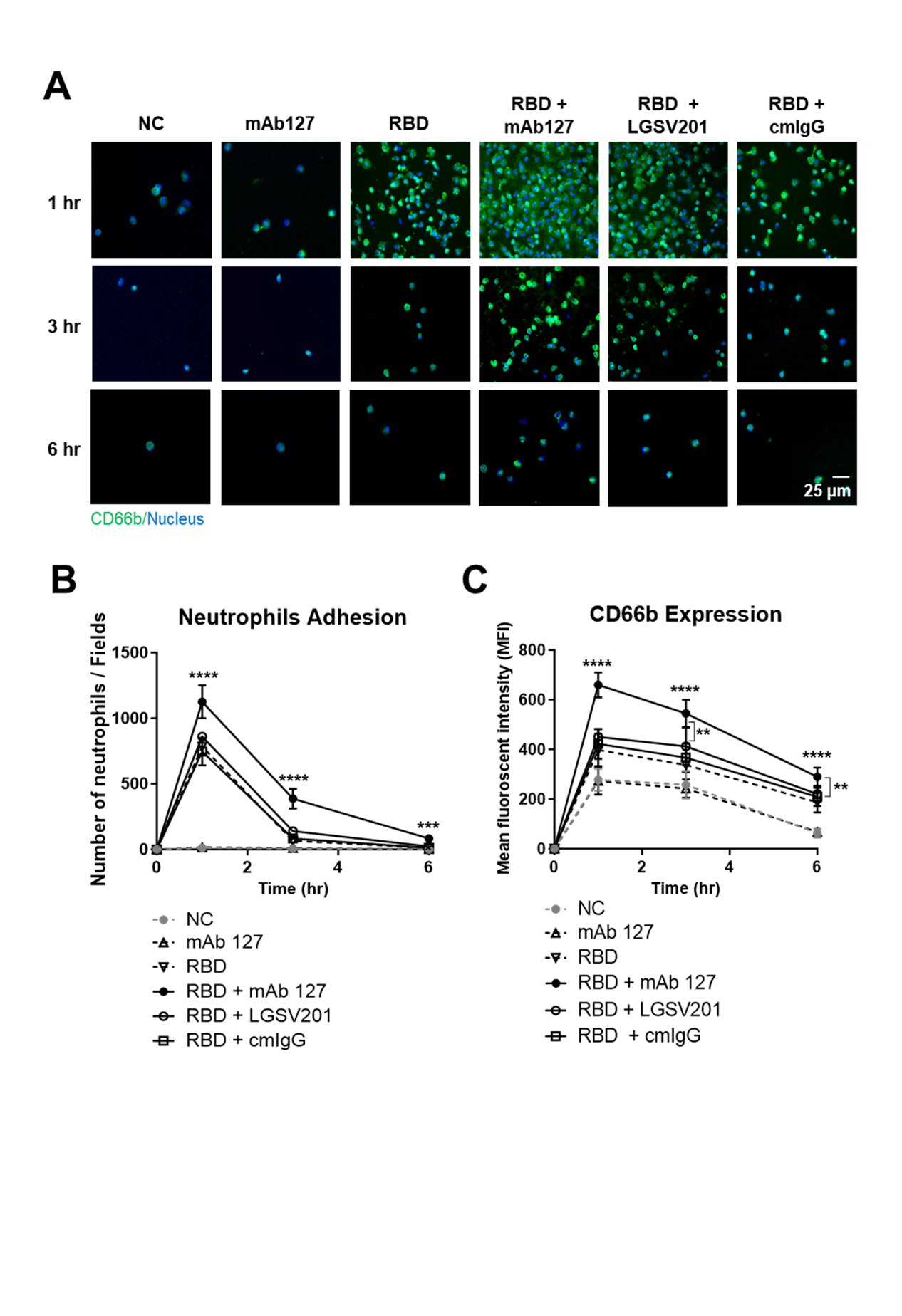
**Fig. S7 CR Abs induced isolated human neutrophil adhesion in the presence of RBD.** Isolated human neutrophils were treated with the recombinant RBD protein in the presence or absence of the indicated antibodies (mAbs 127, LGSV201, or cmIgG). After the indicated time points, (A) the plates were fixed, stained with an anti-CD66b antibody (green) and Hoechst (blue) nuclear stain and then visualized using an immunofluorescence microscope to detect cell adhesion. (B) The number of adhesive cells and (C) mean fluorescence intensity of CD66b expression per cell were quantified by ImageJ. The averages of triplicate cultures ± SD are shown. The views for neutrophil adhesion were randomly selected with 6 pictures from each experiment. Statistical significance was calculated using one-way ANOVA and Tukey’s post hoc test, **p < 0.01, ****p < 0.0001.


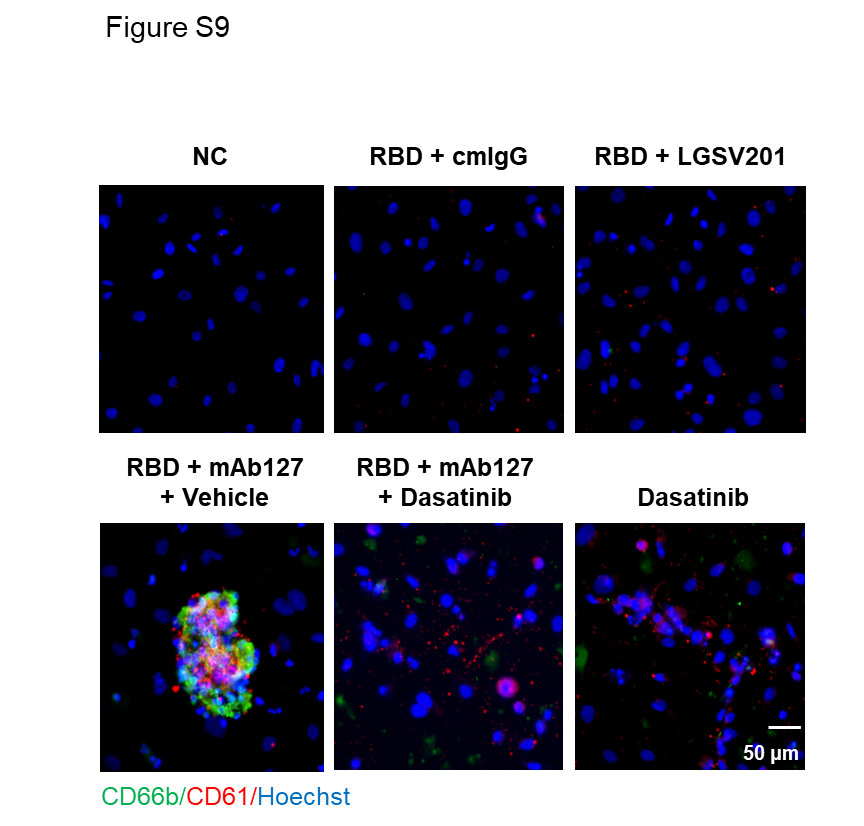
**Fig. S8 SFK signaling is involved in CR Abs-triggered NETosis and thrombosis *in vitro*.** HUVECs were seeded onto poly-L-lysine-coated cover slides for 18-24 h. Isolated human neutrophils and platelets (at a ratio of 20:1) were added onto HUVEC-formed endothelial barriers. After 3 h of stimulation with the RBD protein and the indicated antibodies (mAbs 127, LGSV201, or cmIgG) in the presence or absence of 50 nM dasatinib, the cells were fixed and subjected to immunofluorescence staining with an anti-CD66b antibody (green), anti-CD62P antibody (red) and Hoechst (blue) nuclear stain.

**
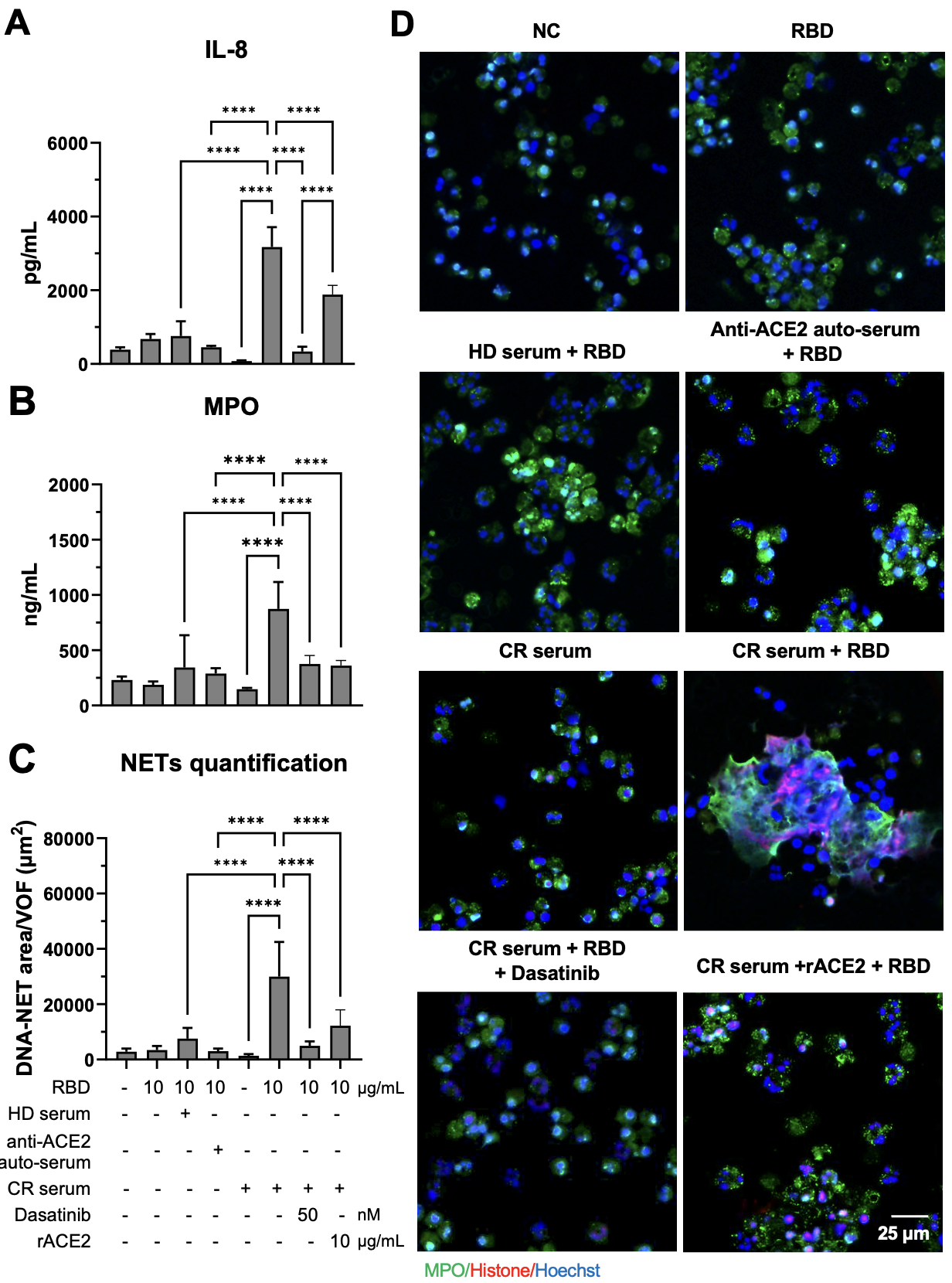
Fig. S9 CR Abs in sera of unvaccinated COVID-19 patients induce neutrophil activation and NETosis in the presence of RBD.** Isolated human neutrophils were preincubated with 1:100 diluted HD serum, anti-ACE2-positive COVID-19 patient serum (anti-ACE2 autoserum), or CR serum in the presence or absence of additional rACE2 (10 μg/mL) or dasatinib (50 nM) as indicated for 30 min. Then, unbound antibodies were removed by centrifugation, and the cells were treated with the RBD protein (10 μg/mL) for 24 h. The supernatants were collected to measure the (A) IL-8 and (B) MPO levels using ELISA kits. (C)(D) The cell suspensions were spun onto a microscope slide by using a cytocentrifuge and were fixed and subjected to immunofluorescence staining with an anti-MPO antibody (green), anti-histone antibody (red) and Hoechst (blue) nuclear stain, and the area of NET formation was quantified by ImageJ. The averages of triplicate cultures ± SD are shown. The views for NET quantification were randomly selected with 9 pictures from each experiment. Statistical significance was calculated using one-way ANOVA and Tukey’s post hoc test, ****p < 0.0001. Bar: 25 μm.
